# Planting long‐lived trees in a warming climate: Theory shows the importance of stage‐dependent climatic tolerance

**DOI:** 10.1111/eva.13711

**Published:** 2024-06-17

**Authors:** Adèle Erlichman, Linnea Sandell, Sarah P. Otto, Sally N. Aitken, Ophélie Ronce

**Affiliations:** ^1^ ISEM, Univ Montpellier, CNRS, IRD Montpellier France; ^2^ Department of Zoology University of British Columbia Vancouver British Columbia Canada; ^3^ Department of Organismal Biology Uppsala University Uppsala Sweden; ^4^ Department of Urban and Rural Development Swedish University of Agriculture Uppsala Sweden; ^5^ Department of Forest and Conservation Sciences University of British Columbia Vancouver British Columbia Canada

**Keywords:** assisted gene flow, climate change, complex life cycles, forestry, local adaptation, seed sourcing

## Abstract

Climate change poses a particular threat to long‐lived trees, which may not adapt or migrate fast enough to keep up with rising temperatures. Assisted gene flow could facilitate adaptation of populations to future climates by using managed translocation of seeds from a warmer location (provenance) within the current range of a species. Finding the provenance that will perform best in terms of survival or growth is complicated by a trade‐off. Because trees face a rapidly changing climate during their long lives, the alleles that confer optimal performance may vary across their lifespan. For instance, trees from warmer provenances could be well adapted as adults but suffer from colder temperatures while juvenile. Here we use a stage‐structured model, using both analytical predictions and numerical simulations, to determine which provenance would maximize the survival of a cohort of long‐lived trees in a changing climate. We parameterize our simulations using empirically estimated demographic transition matrices for 20 long‐lived tree species. Unable to find reliable quantitative estimates of how climatic tolerance changes across stages in these same species, we varied this parameter to study its effect. Both our mathematical model and simulations predict that the best provenance depends strongly on how fast the climate changes and also how climatic tolerance varies across the lifespan of a tree. We thus call for increased empirical efforts to measure how climate tolerance changes over life in long‐lived species, as our model suggests that it should strongly influence the best provenance for assisted gene flow.

## 1 INTRODUCTION

There is strong evidence for the local adaptation of many populations to their historical climate (Alberto et al., 2013; Lortie & Hierro, 2022; Savolainen et al., 2007; Wadgymar et al., 2022). Current anthropogenic global warming is, however, disrupting climatic conditions (Intergovernmental Panel on Climate Change [IPCC], 2021), decoupling populations from the climate to which they were historically adapted (Benito‐Garzón et al., 2018; Bontrager & Angert, 2019; Gauli et al., 2022; Hanewinkel et al., 2013; Thomas et al., 2004). Climate change‐related maladaptation could thus lead to, or exacerbate, population declines (Frank et al., 2017), especially when combined with other pressures faced by biodiversity (Maxwell et al., 2016). While local adaptation to new climatic conditions may be restored with time due to natural evolutionary and ecological processes, such as adaptive phenotypic changes (Bell, 2017; Yeh & Price, 2004) and range shifts (Chen et al., 2011; Freeman et al., 2018; Lenoir & Svenning, 2015), these natural processes may be insufficient in species with low evolutionary potential (Shaw & Etterson, 2012), long generation time (e.g., Cotto et al., 2017), maladaptive phenotypic plasticity (Diamond & Martin, 2021; Fox et al., 2019) or poor dispersal ability (Leimu et al., 2010).

In circumstances where life cycle attributes severely limit natural dispersal, human‐assisted migration has been proposed to accelerate population adaptation to climate change, either as a conservation strategy or to maintain productivity for economically important species (Isabel et al., 2020). Assisted migration, also called assisted gene flow when focusing on movements within the historical range of populations (Aitken & Whitlock, 2013), uses the translocation of pre‐adapted genotypes in anticipation of or in response to environmental change (Aitken & Bemmels, 2016). Risks associated with these practices (Aitken & Whitlock, 2013; Ricciardi & Simberloff, 2009; Sáenz‐Romero et al., 2021; Weeks et al., 2011) include maladaptation, outbreeding depression, gene swamping, hybridization, pathogen introduction and invasive spread. We still have few tools to predict the efficiency of such interventions in mitigating the negative consequences of climate change and their optimal design. Our aim here is to develop simple theoretical arguments contributing to a better understanding of trade‐offs affecting assisted migration in long‐lived species. Because the risks are thought to be smaller in the case of assisted gene flow (Aitken & Whitlock, 2013), we will focus on scenarios mimicking translocations within a species range. Our model, however, also helps understand trade‐offs affecting translocations beyond the range limits.

Assisted gene flow is particularly discussed in the context of forestry practices (Aitken & Whitlock, 2013). Indeed, the long generation time and limited dispersal capabilities resulting from their prolonged sessile phase make long‐lived trees particularly vulnerable to the effects of climate change (e.g., Brodribb et al., 2020; Gougherty et al., 2021). Most temperate and boreal forest tree species are locally adapted to climate (Leites & Benito Garzón, 2023) and already suffer negative consequences of climate change (e.g., Abram et al., 2021; Davis et al., 2019; Forzieri et al., 2022; Hartmann et al., 2022; Hellmann et al., 2008; Sáenz‐Romero et al., 2020; Stevens‐Rumann et al., 2018; Sturrock et al., 2011). The vulnerability and decline of tree populations is of interest because trees provide numerous ecosystem services (Brockerhoff et al., 2017), with important ecological (e.g., providing food resources and habitat for many other species (Yu et al., 2023), carbon storage, microclimate regulation), economic (Fraga et al., 2020), societal (Esperon Rodriguez et al., 2022) and cultural roles (Agnoletti & Santoro, 2015; Lewis & Sheppard, 2005; Trigger & Mulcock, 2005).

To implement assisted gene flow, one needs to identify seed sources that maximise the survival and productivity of the transplanted individuals in the future, based on projected climate change. In forestry, the population used for seed sourcing is referred to as a “provenance”, specifically in reference to the geographical location (Aitken & Bemmels, 2016). Traditional seed‐sourcing strategies use transfer functions and provenance trials to identify provenances best adapted to a plantation site (e.g., O'Neill et al., 2017; Risk et al., 2021; Thomson et al., 2009). Historically, the use of local provenances was widely recommended for reforestation (O'Neill & Gómez‐Pineda, 2021). Managers, however, are increasingly being advised to anticipate near‐future climate change when selecting seed sources for plantations (e.g., Aitken & Bemmels, 2016; Benito‐Garzón et al., 2018; Girard et al., 2022; Sáenz‐Romero et al., 2021; St. Clair et al., 2022; Whittet et al., 2016). For instance, in British Columbia, Canada, a recently adopted policy advises that planted populations be adapted to the expected future climate at the plantation site (O'Neill et al., 2017). Several assisted gene flow experiments are already underway (e.g., McDonald et al., 2021; Prieto‐Benıtez et al., 2021; Young et al., 2020).

Only a few recent models have, however, begun to investigate how different assisted gene flow strategies can accelerate adaptation (DeFilippo et al., 2022; Grummer et al., 2022; Quigley et al., 2019), and even fewer have attempted to quantify the demographic consequences of assisted gene flow and its impact on population persistence and function (e.g., Bay et al., 2017; Kelly & Phillips, 2019; Kuparinen & Uusi‐Heikkilä, 2020). Many organisms targeted for assisted gene flow, such as trees or corals, are long‐lived and have complex life cycles: life cycle stages (e.g., seed, seedling, sapling, pole, mature tree and senescent stages) differ in their tolerance to climatic variables (Du et al., 2019; Mašek et al., 2021; Pompa‐Garcıa & Hadad, 2016), and different individuals may spend different amounts of time in each stage before growing to the next (Jackson et al., 2009). Yet, we have little insight into how complex life cycles may affect optimal assisted gene flow strategies. In very long‐lived organisms, a trade‐off occurs when climate changes during an individual's lifetime: individuals will on average experience colder temperatures early in their lives and warmer temperatures later. The alleles conferring good performance may then be different at the beginning and end of life. In particular, there is concern that warm‐adapted provenances that will perform well in the future could still suffer from damage due to colder temperatures while juvenile (Sebastian‐Azcona et al., 2019). Previous theoretical studies have suggested that it will be challenging for long‐lived species with complex life cycles to adapt to a changing climate, not only because their long generation time slows down evolution but also because of trade‐offs in adaptation to climate change between different life stages (Cotto et al., 2019; Cotto & Chevin, 2020; Marshall et al., 2016; Marshall & Connallon, 2022).

In organisms with a complex life cycle, different stages typically make different contributions to the growth of the population (Caswell, 2000). In long‐lived trees, for instance, individuals in young stages with high mortality typically have a low contributions to population dynamics compared to older individuals. In reintroduction or reinforcement programs, releasing individuals with high reproductive value and contribution to population growth can increase the success of the program (Sarrazin & Legendre, 2000). In assisted gene flow programs, however, it is unknown whether similar reasoning applies: is it optimal to select seed sources that will be best adapted at the stage with the highest contribution to population growth, i.e., when fully grown? Or is it optimal to source seeds that provide the most protection during the life stages that are least tolerant to climate change? Some studies suggest that young stages tend to be less tolerant to stress than older stages (e.g., Black & Bliss, 1980; Kueppers et al., 2017; Munier et al., 2010). For example, in British Columbia, the current recommendation is to plant populations adapted to the expected future climate at one quarter of the rotation time (i.e., time between planting and harvest), when trees are still relatively young, to account for the lower tolerance of seedlings to maladaptation (O'Neill et al., 2017). These rules of thumb, however, lack a strong theoretical foundation, and we do not know how the choice of the best provenance depends on the exact life cycle of the tree, the speed of climate change and the impact it has on different life stages.

The aim of this paper is, therefore, to fill this gap by theoretically exploring the effect of (1) changes in tolerance to climate over the lifetime of trees and (2) variation in life history (i.e., variation in stage‐specific vital rates) on the choice of the best provenance for assisted gene flow. To this end, we use a simple scenario that describes the persistence of a single cohort of long‐lived trees in a warming environment. This scenario approximates even‐aged forest management, where seedlings of the same age are planted after a clearcut and harvested a number of decades later (Puettmann et al., 2015). It will serve as a baseline for future exploration of more complex forestry scenarios. To understand what determines the best provenance for maximizing the survival of a cohort of trees under climate change, we extend the analytical work of Cotto and Chevin (2020), who used an age‐structured model to examine the evolution of a quantitative trait in organisms that undergo multiple episodes of selection in a changing environment. We instead consider a stage‐structured model that better represents the life history of species like trees, for which size more than age determines the survival prospects. We derive analytical predictions for the best provenance to maximize cohort survival under climate change. To develop a better understanding, we first illustrate these mathematical predictions in the simplest case, where there are only two stages in the life cycle (juveniles and adults), exploring how the time spent in each stage and the difference in climatic tolerance across stages affect the choice of the best provenance. We then parameterize our model with empirically measured life histories for 20 long‐lived species. We check that the qualitative conclusions derived in the two‐stage case hold when the number of stages increases and for various tree life histories. In addition, we explore the effect of changes in various assumptions to assess the robustness of our results to: (i) the change in thermal tolerance across life, (ii) the rate of environmental warming, (iii) random interannual variation in temperature and (iv) variation in thermal tolerance among individuals from the same cohort. We compare the potential benefits of implementing assisted gene flow predicted by our model for different choices for seed sourcing: planting only seeds from the local provenance, planting the best provenance predicted by our model, or using a simple rule of thumb to match seed provenance to the future climate in the plantation site, such as in British Columbia (O'Neill et al., 2017).

## 2 METHODS

### 2.1 Biological scenario

Given a single cohort of trees planted at *t* = 0 and harvested after *H* years under environmental warming, we aim to optimize the seed source (“provenance”) considering the trade‐off between current climate adaptation for seedlings and future climate adaptation for older trees. Trees in our model have a complex life cycle spanning *n* discrete stages, each with a different tolerance to climate. The seed source that yields the highest number of surviving trees in the last stage *n* after *H* years will be denoted as having the best provenance. Note that throughout this study, we use the term “harvest” to quantify the final size of the cohort. Maximizing the size of a cohort (i.e. the number of individuals reaching peak reproductive stage) also achieves conservation goals when individuals are not harvested.

For the sake of illustration, we characterize each provenance by the mean annual temperature that would maximize the survival of trees from that provenance in a constant climate (their thermal optimum, θ). This choice is motivated by the fact that mean annual temperature is frequently used to characterize the climatic niche of different provenances in experimental plantations (e.g., Pedlar, McKenney, Lu, 2021; Pedlar, McKenney, Lu, Thomson, 2021; Wang et al., 2006). Our model could, however, be similarly applied to any climatic variable or combination of climatic variables affecting life‐history traits and characterizing the climatic niche of the different provenances (θ would then be a vector with all relevant niche dimensions, e.g., showing the optimal position along precipitation and temperature gradients). Each cohort is made up of trees from a single provenance, all of which share a constant thermal optimum throughout their life. The thermal niche width of this cohort is, however, allowed to vary across life stages, reflecting different sensitivities to temperature change. To evaluate the robustness of our predictions, we later relax the assumption that all individuals in the cohort have the same thermal optimum. Individuals from the same provenance may indeed vary in their response to temperature, and seed sourcing strategies may involve mixing different provenances (Breed et al., 2018; Whittet et al., 2016). These numerical results are presented in Supplementary material S1.

We assume that the annual survival rate declines as individuals are exposed to temperatures deviating from their thermal optimum. For mathematical convenience and building upon previous theoretical quantitative genetic models of adaptation to variable environments (Cotto & Chevin, 2020), we more precisely define the survival rate in stage *i* (*s*_*i*_) as a Gaussian function of the current temperature $T_{t}$ in year t, with $s_{i,\max}$ being the maximal survival rate in stage i:

(1) $$s_{i}\left(\theta ,T_{t}\right)=s_{i,\max}\exp\left(-\frac{{\left(\theta -T_{t}\right)}^{2}}{2\omega_{i}^{2}}\right)$$

An individual has the highest probability of surviving when the yearly temperature matches its thermal optimum $T_{t}=\theta$. The parameter $\omega_{i}$ scales the thermal tolerance of an individual in stage i: according to the above expression, when the temperature deviates from its optimal temperature (θ) by $\omega_{i}$ degrees, the survival rate is reduced by a factor $1-e^{-\frac{1}{2}}\approx 40\%$.

### 2.2 Stage structure dynamics

**FIGURE 1:**
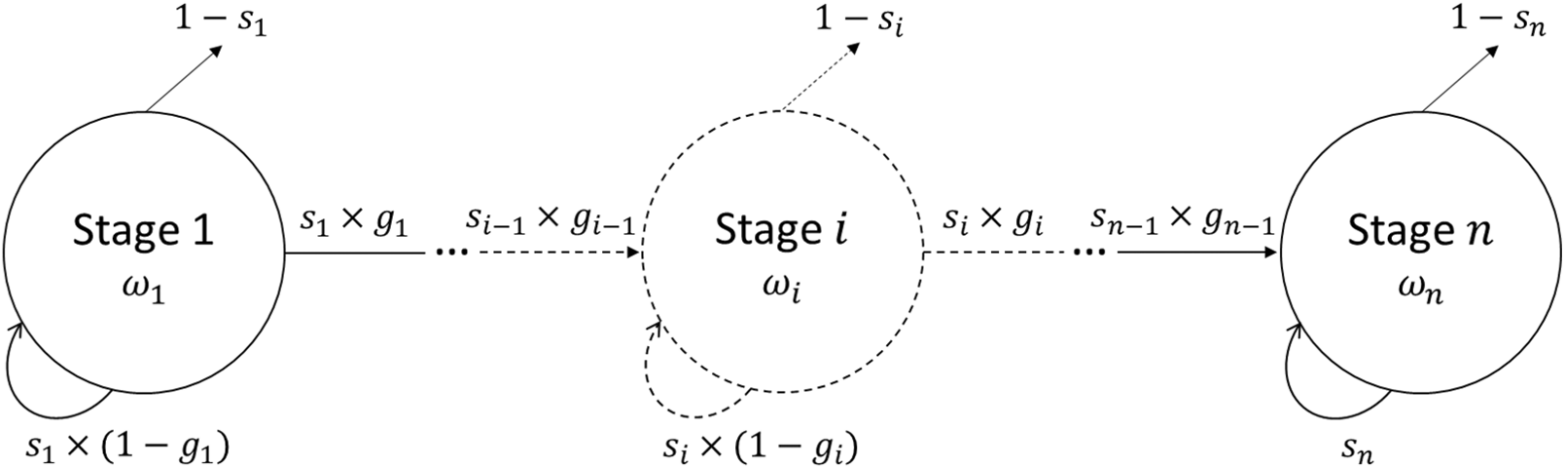
Life cycle illustration with *n* stages. Parameters: *s*_*i*_ is the annual probability of survival, *g*_*i*_ is the annual probability of growing to the next stage, and *ω*_*i*_ is the temperature tolerance in stage *i*.

We track the number of individuals in a stage‐structured population under this scenario (Figure 1). The transition probabilities between stages, from year to year, are described by an *n*‐by‐*n* matrix M, and the number of individuals in each stage is stored in a vector $N_{t}$. Each year, individuals can either remain in their current stage if they survive and do not grow sufficiently to transition (with probability $s_{i}\left(\theta ,T_{t}\right)\times \left(1-g_{i}\right)$), die (with probability $1-s_{i}\left(\theta ,T_{t}\right)$), or transition to the next stage if they survive and grow (with probability $s_{i}\left(\theta ,T_{t}\right)\times g_{i}$), where $g_{i}$ is the probability of growing to stage i+1 conditional on survival. We only allow transitions to occur to the same or the following stage, i.e., we assume trees neither shrink nor skip stages. Hence, at the next time step t+1, the number of individuals in each stage is:

(2) $$N_{t+1}=M\left(\theta ,T_{t}\right)\cdot N_{t}$$

We do not explicitly consider the effects of density‐dependent competition on survival, but assume that empirical estimates of survival and growth rates in natural populations that we use to parameterize the model already integrate the effect of competition and were measured at densities relevant to our forestry scenarios. Individuals can remain in a stage for several years, and different individuals can stochastically grow to the next stage at different ages. We denote by $a_{i}$ the age at which an individual makes the transition from stage i to stage i+1, where $i\in \left[1,\ldots ,n-1\right]$. We define $\vec{a}={\left[a_{1},\ldots ,a_{n-1}\right]}^{T}$ as the individual's life history, containing the age at which an individual transitioned to each stage, given that it reached the last stage before harvest, and $p\left(\vec{a}\right)$ as the fraction of surviving individuals at harvest with this realized life history.

We also investigate a scenario where both growth $\left(g_{i}\right)$ and survival $\left(s_{i}\right)$ rates depend on temperature, which shows very similar results to the case discussed in the main text (Supplementary material S2; Figure S3).

### 2.3 Analytical predictions

We derive an expression for the expected number of mature trees at harvest time, $N_{H}$, given an initial cohort of $N_{0}$ seedlings from a provenance with a thermal optimum θ. We begin by assuming that all trees share a particular life history $\vec{a}$. Indeed, when the time spent in each stage is fixed, we can apply the age‐structured results of Cotto and Chevin (2020) for multiple rounds of Gaussian selection to a single cohort. At harvest, the pool of remaining individuals is formed by the individuals that have survived each stage and reached the last:

(3) $$E\left[N_{H}|\vec{a}\right]=N_{0}\prod_{t=1}^{a_{1}}s_{1}\left(\theta ,T_{t}\right)\times \cdots \times \prod_{t=a_{i-1}+1}^{a_{i}}s_{i}\left(\theta ,T_{t}\right)\times \cdots \times \prod_{t=a_{n-1}+1}^{H}s_{n}\left(\theta ,T_{t}\right)$$

Because Equation 3 is a product of Gaussian functions, we can use the result of Cotto and Chevin (2020) to write the expected number of remaining individuals as:

(4) $$E\left[N_{H}|\vec{a}\right]=N_{0}\;s_{\mathrm{tot},\max}\left(\vec{a}\right)\exp\left(-\frac{{\left(\theta -T_{\mathrm{tot}}\left(\vec{a}\right)\right)}^{2}}{2\;\omega_{\mathrm{tot}}^{2}\left(\vec{a}\right)}\right)$$

with

(5) $$\omega_{\mathrm{tot}}^{2}\left(\vec{a}\right)={\left(\frac{a_{1}}{\omega_{1}^{2}}+\cdots +\frac{a_{i}-a_{i-1}}{\omega_{i}^{2}}+\cdots +\frac{H-a_{n-1}}{\omega_{n}^{2}}\right)}^{-1}$$

(6) $$T_{\mathrm{tot}}\left(\vec{a}\right)=\sum_{t=1}^{a_{1}}\frac{\omega_{\mathrm{tot}}^{2}\left(\vec{a}\right)}{\omega_{1}^{2}}T_{t}+\ldots +\sum_{t=a_{i-1}+1}^{a_{i}}\frac{\omega_{\mathrm{tot}}^{2}\left(\vec{a}\right)}{\omega_{i}^{2}}T_{t}+\ldots +\sum_{t=a_{n-1}+1}^{H}\frac{\omega_{\mathrm{tot}}^{2}\left(\vec{a}\right)}{\omega_{n}^{2}}T_{t}$$

(7) $$\begin{array}{l} s_{\mathrm{tot},\max}\left(\vec{a}\right)=\left(s_{1,\max}^{a_{1}}\times \ldots \times s_{i,\max}^{a_{i}-a_{i-1}}\times \ldots \times s_{n,\max}^{H-a_{n-1}}\right)\\ \exp\left(\frac{T_{\mathrm{tot}}{\left(\vec{a}\right)}^{2}}{2\omega_{\mathrm{tot}}^{2}\left(\vec{a}\right)}-\sum_{t=1}^{a_{1}}\frac{T_{t}^{2}}{2\omega_{1}^{2}}-\ldots -\sum_{t=a_{i-1}+1}^{a_{i}}\frac{T_{t}^{2}}{2\omega_{i}^{2}}-\ldots -\sum_{t=a_{n-1}+1}^{H}\frac{T_{t}^{2}}{2\omega_{n}^{2}}\right) \end{array}$$

According to Equation 4, the probability that an individual with a life history $\vec{a}$ survives until harvest is maximal and equal to $s_{\mathrm{tot},\max}\left(\vec{a}\right)$, if its thermal optimum θ matches the realized temperature experienced across its lifespan $T_{\mathrm{tot}}\left(\vec{a}\right)$, weighting the temperature at each age by the thermal tolerance of the stage corresponding to that age as in Equation 6. The “cumulative thermal tolerance” experienced by an individual with a growth trajectory $\vec{a}$ across its lifespan is measured by $\omega_{\mathrm{tot}}\left(\vec{a}\right)$, which can be computed as $\omega_{\mathrm{tot}}^{2}\left(\vec{a}\right)=\frac{{\tilde{\omega }}^{2}}{H}$, where ${\tilde{\omega }}^{2}$ is the harmonic mean of the squared thermal tolerances experienced by an individual throughout its life, as given by Equation 5. If temperature tolerance is constant throughout life (i.e., $\omega_{i}^{2}$ is constant at $\omega^{2}$), then $\omega_{\mathrm{tot}}^{2}\left(\vec{a}\right)=\frac{\omega^{2}}{H}$. In this case, the best provenance has a thermal optimum equal to the midpoint temperature experienced during the lifespan of a tree (i.e., when $\theta =T_{\mathrm{tot}}\left(\vec{a}\right)=\frac{1}{H}\sum_{t=1}^{H}T_{t}$; Cotto & Chevin, 2020).

The above result applies only to stage‐structured populations when there is a fixed amount of time between stages, in which case the stage‐structured model is equivalent to an age‐structured model. More generally, because individuals of the same cohort will vary stochastically in the time they spend in each stage, we must consider all possible life histories ($\vec{a}$) to estimate the number of remaining individuals at harvest.

Accounting for all possible life histories, the pool of individuals that survive and grow to harvestable size by time H is expected to be:

(8) $$E\left[N_{H}\right]=\sum_{\vec{a}}p\left(\vec{a}\right)\;E\left[N_{H}|\vec{a}\right]$$

where $p\left(\vec{a}\right)$ is the probability of the life history $\vec{a}$ conditional on survival until harvest, given by $p\left(\vec{a}\right)=\prod_{i=1}^{n-1}{\left(1-g_{i}\right)}^{a_{i}-a_{i-1}-1}\;g_{i}$ with $a_{0}=0$.

For the sake of illustration, we explore the prediction of this analytical model in the simple case where there are only two stages (e.g., adults and juveniles). In this simple case, the life history of a surviving individual can be uniquely characterized by the age at transition between the two stages (e.g., age at maturity). We also assess whether predictions about the best provenance, using Equation (4) and the average age at transition in the cohort, provide a good approximation for what happens in the more complex stage‐structured case where individuals may differ in their life history. We numerically look for the best provenance maximizing $E\left[N_{H}\right]$ when integrating across all possible growth trajectories, using Equation (8). We also compute the mean age of maturation a among survivors at harvest, integrating across all possible life histories for a given life cycle and use that value to compute $T_{\mathrm{tot}}\left(a\right)$ as in Equation (6). We compare the thermal optimum of the best provenance predicted by the two methods. To generate variation in the mean age of maturation among survivors at harvest, we repeat these calculations, drawing 500 values of *g*, $s_{1,\max}$ and $s_{2,\max}$ from uniform distributions between 0.01 and 0.99.

### 2.4 Simulations with vital rates for twenty long‐lived tree species

We now turn to more complex life cycles, with more than two stages, and explore how the best provenance varies as a function of life‐history parameters. We extract empirically measured transition matrices (**M**) for long‐lived tree species from the COMPADRE database (Salguero‐Gómez et al., 2015) and perform simulations using Equation (2) for these examples. It should be noted that the transition matrices gathered are quite variable in the way that they categorize life stages, both in the definition and number of stages (see details in Supplementary material S4). We assume that these trees are adapted to the climatic conditions in which they are currently growing. This allows us to estimate $s_{i,\max}$ and $g_{i}$ from the transition rates between stages in the empirical matrices by assuming that the vital rates are at their optima.

In our model, we assume that different provenances of the same species have different climatic optimum, but they have the same vital rates as long as they are each at their optimal temperature. For each species, we investigate how different provenances perform in the face of climate change. We vary the thermal optimum of the provenance θ between the current temperature at the planting site $T_{0}$ to $T_{0}+4^{{}^{\circ}}C$, in increments of 0.01 degrees. The thermal optima of the provenances span the range of temperatures predicted over the lifespan of the trees for all warming scenarios considered (see below and Table 1). Each simulation iterates Equation (2) for H years and computes the number of individuals that have reached the last stage $\left(n\right)$ at harvest, $N_{H}$. We then numerically look for the provenance whose thermal optimum $\left(\theta \right)$ results in the largest number of survivors, called the best provenance. Stand rotation periods in forestry vary widely among species, climates and management practices, from less than 12 years for some fast‐growing tropical species to more than 150 years for some oak stands (Bauhus et al., 2009). In the simulations, we consider a mid‐range rotation period, with harvest occurring after H=60 years (some simulations were run with a longer rotation time in Supplementary Material S6).

**TABLE 1:** Temperature warming scenarios.

Scenario	IPCC scenario	Average warming in 60 years
Control	–	+0°C
Intermediate	SSP2‐4.5	+1.7°C
High	SSP3‐7.0	+2.6°C
Very high	SSP5‐8.5	+3.3°C

Simulations are run in Wolfram *Mathematica* (Version 12.0.0.0), while numerical searches for the best provenance are conducted in R (Version 4.2.3). Both scripts can be found in Supporting Information (Code S1, S2 and S3). We checked that the simulations, described above, and the analytical predictions from Equation 8 yield identical results for a two‐stage life cycle (Supplementary material S3; Figure S4).

#### 2.4.1 Species selection

We filter the COMPADRE database by OrganismType to obtain demographic parameters for tree species. As data on local adaptation for tropical tree species are scarcer and the impact of climate change is less straightforward (Aitken & Bemmels, 2016; IPCC, 2021), we also filter by Ecoregion to keep only non‐tropical trees. The following ecoregions are retained in our data set: Temperate Broadleaf and Mixed forests (TBM), Temperate Coniferous Forests (TCF), Boreal forests and taiga (BOR), Montane grasslands and shrublands (MON), Temperate grasslands, savannas and shrublands (TGS), Mediterranean forests, woodlands and scrubs (MED). We obtained 47 species in total. From these, we selected 19 species that had complete matrices and for which individuals had reached the last stage before the time of harvest (set to 60 years). When several matrices are available for a species, we keep those that pool all years, locations, or treatments to consider typical growth trajectories. We also add a matrix for American beech (*Fagus grandifolia*), originally described in Harcombe (1987). To test that our qualitative conclusions hold for trees with slower growth and thus longer harvest times, we use a harvest time of 100 years and three matrices of species that reach the last stage after 60 years and before 100 years (see Supplementary Material S6). Details about the matrices used can be found in Supporting Information (Data S1).

#### 2.4.2 Modelling the change in tolerance across the lifespan of trees

The breadth of thermal tolerance in trees is typically estimated through translocation and provenance tests in common gardens (Aitken et al., 2008; Aitken & Bemmels, 2016; Mátyás, 1996; Schmidtling, 1994; Sork et al., 2013; Wang et al., 2010). We unfortunately failed to find such data on survival as a function of climatic distance for any of the species that we have selected in the COMPADRE database. Consequently, we cannot estimate thermal tolerance and how it changes across life‐history stages for any of the species with demographic data. In the absence of such data, we explore the theoretical impact of changes in thermal tolerance on harvest using a simple hypothetical linear model for variation in thermal tolerance with stage: $\omega_{i}^{2}=\omega_{1}^{2}\;\left(1+b\;\left(i-1\right)\right)$. A positive or negative value of b corresponds to a scenario where older or younger trees are more tolerant to temperature changes, respectively.

We investigate the effect of both scenarios of increasing or decreasing tolerance with age with our analytical model for a life cycle with two stages. Because this analysis shows the results to be quite symmetrical and because younger trees are expected to be less tolerant than fully grown trees, we focus only on cases with positive b in the simulations. We hold the cumulative thermal tolerance $\omega_{\mathrm{tot}}\left(\vec{a}\right)$ constant across species and also within species when we contrast different scenarios for change in thermal tolerance across stages: we then vary the value of b, which, for a constant $\omega_{\mathrm{tot}}\left(\vec{a}\right)$, determines the value of the thermal tolerance of the first stage ($\omega_{1}$).

To calculate stage‐specific tolerances for each species while holding the cumulative thermal tolerance $\omega_{\mathrm{tot}}\left(\vec{a}\right)$ constant, we approximate the typical life history $\vec{a}$ of each species by calculating the expected time spent in each stage i from the fundamental matrix F, such that $F={\left(I-M\right)}^{-1}$ (see Caswell et al., 2018). The expected age of maturation from stage i to stage i+1 is $a_{i}=\sum_{j=1}^{i}F_{1,j}$. These values are rounded to the next integer to obtain a typical life history for that species. We then use such values of $a_{i}$ and replace the stage‐specific tolerances by $\omega_{i}^{2}=\omega_{1}^{2}\;\left(1+b\;\left(i-1\right)\right)$ in Equation 5, to obtain the value of $\omega_{1}$ as a function of b and $\omega_{\mathrm{tot}}\left(\vec{a}\right)$ for each species. This method to compute the average ages at transition between stages is not conditional on the survival of individuals to the last stage, so the life history used to estimate $\omega_{\mathrm{tot}}\left(\vec{a}\right)$ may differ in some cases from the realized life history of harvested individuals. We find that, as a result, the effective cumulative tolerance of species is on average 0.1°C higher than the theoretical cumulative tolerance, with 95% of effective cumulative tolerance values less than 0.5°C above the theoretical cumulative tolerance initially set.

In the different figures, we represent the extent of change in thermal tolerance during ontogeny by the ratio of thermal tolerance in the last and first stages $\omega_{n}/\omega_{1}$. Note that varying this ratio within each species helps evaluate how much the choice of the best provenance depends on the (unknown) change in thermal tolerance across life. This ratio is actually likely to vary between species, in particular because of the variable definition of the first stage in the different tree life cycles in the COMPADRE database.

We replicate these simulations for 3 different values of the cumulative thermal tolerance $\omega_{\mathrm{tot}}\left(\vec{a}\right)$: 2, 3.5, and 5°C. The choice of order of magnitude for these values is inspired by the results of Rehfeldt et al. (1999). In that study, one‐year‐old seedlings of *Pinus contorta* were planted, and survival was estimated at age 20 for two different provenances, yielding an estimate of the cumulative thermal tolerance of $\approx 2.75^{{}^{\circ}}C$ over 20 years. If thermal tolerance did not vary with age, this would predict a cumulative tolerance over 60 years of <1°C according to Equation 5. If tolerance increases with age, as we suspect, we, however, expect the cumulative tolerance over 60 years to exceed the latter estimation. The chosen values contrast situations where the climatic niche of trees is relatively narrow, intermediate, or wide, while being not widely unrealistic given extant evidence on survival tolerance to temperature in forest trees.

#### 2.4.3 Scenarios of environmental change

To better understand the interactions between the rate of warming and the species life cycle affecting the choice of the best provenance, we simulate scenarios where each species is submitted to the same average warming trend. We first consider a linear increase in mean annual temperature, at speed k, such that $T_{t}=T_{0}+\mathrm{kt}$ (average warming in 60 years in Table 1), where $k=\frac{T_{H}-T_{0}}{H}$.

We parameterize the rate of change k in mean annual near‐surface air temperature using the latest climate change projections available, the CMIP6 climate models, available in the Intergovernmental Panel on Climate Change (IPCC) WGI Interactive Atlas. The projected mean increase in annual mean air temperature for the WGI reference regions can be downloaded as GeoTIFF maps from the compilation of 34 different climate models at https://interactive‐atlas.ipcc.ch. We consider three temperature warming scenarios estimated in the IPCC Sixth Assessment Report (Table SPM.1; IPCC, 2021): SSP2‐4.5, SSP3‐7.0 and SSP5‐8.5. We choose to use the period 1995–2014 as a baseline and the projected temperatures in 2081–2100. To approximate the temperature increase in the regions where the species occur, instead of using the global average, we retrieve the predicted increase in mean annual air temperature at the geographic coordinates given for each demographic transition matrix that we downloaded from COMPADRE (see Supplementary Material S5; Figure S5). From this, we compute the predicted rate of increase in mean annual air temperature at each species location per year and average this value over all locations to obtain k. We also use a scenario with no warming as a control.

To test the robustness of the results to random variation in mean annual temperature around that trend, we also run simulations where each year the temperature fluctuates around the intermediate warming trend (+1.7°C). Each year, the value of the annual mean temperature is randomly drawn from a normal distribution centred on the value of $T_{t}$, which increases linearly over time.

We consider three temperature fluctuation scenarios based on the estimates of Olonscheck et al. (2021). Inspired by their reported historical global average of observed and simulated local standard deviations of 0.44 and 0.47°C, we explore a scenario, which we call moderate fluctuations, with the standard deviation around the mean annual temperature of 0.5°C, assuming no major change in the future in the extent of year‐to‐year fluctuations in temperature. Different climate models examined in Olonscheck et al. (2021) consistently predict decreased year‐to‐year fluctuations in mean temperature at high latitudes and increased fluctuations in the tropics by the end of the century, but the predictions do not converge in many parts of the world. Given this high uncertainty, we also explore two scenarios with 30% weaker or 30% stronger fluctuations (corresponding to standard deviations of 0.35 and 0.65°C, respectively).

For the sake of illustration, we run the simulations with fluctuations around the mean annual temperature for two species with contrasting life histories (*C. decurrens* and *A. concolor*, both with n=5 stages). We replicate each scenario 100 times to explore different temperature trajectories and determine the best provenance for each replicate. We here simulate cohort survival for a broader range of provenances thermal optimums (ranging from −5°C below to 5°C above the current local temperature) to account for instances of low temperatures that may occur stochastically in some runs. We consider two cases: constant thermal tolerance over the tree's lifespan $\left(\omega_{5}/\omega_{1}=1\right)$ and lower temperature tolerance in young trees compared to old ones $\left(\omega_{5}/\omega_{1}=9\right)$.

## 3 RESULTS

### 3.1 Analytical results in a two‐stage case

**FIGURE 2:**
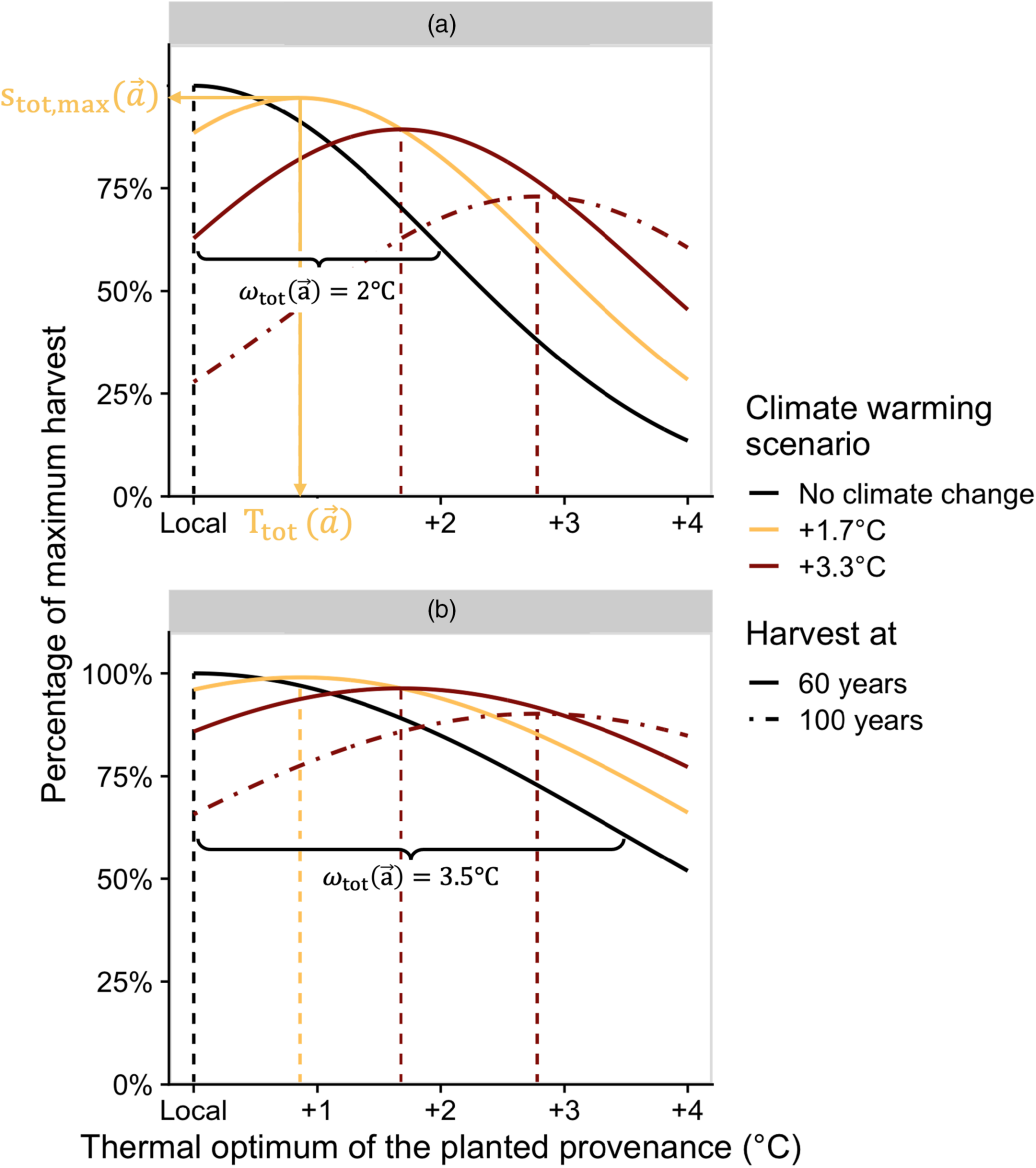
The potential loss in harvest from planting suboptimal provenances is greater if trees have low cumulative tolerance (panel a vs. panel b), especially under more severe climate warming scenarios in 60 years (coloured curves), and if the time before harvest is longer (dot‐dashed curves versus solid curves). For each scenario, the dashed vertical lines represent the thermal optimum of the best provenance. In panel a, the best provenance to plant (*T*_tot_($\vec{a}$)), the maximal number of surviving trees in the cohort (*s*_tot,max_($\vec{a}$)) and the cumulative thermal tolerance (*ω*_tot_($\vec{a}$)) are indicated for the climate warming scenario of +1.7°C (yellow curve). The latter is also indicated on the same curve in panel b, for comparison. For the scenario of harvest after 100 years, the very high warming scenario (of 3.3°C in 60 years) gives a warming of 5.5°C in 100 years. The curves were produced with the two‐stage analytical result with the following parameters: *s*_1_ = 0.61, *s*_2_ = 0.98 and *g* = 0.012.

To better understand the impact of changing thermal tolerance across the lifespan, we first explore a simplified two‐stage case with juvenile and mature trees, for which general analytical predictions are illustrated. While a two‐stage model is a gross simplification of the life cycle of trees, this simple case has the advantage that life history impacts the best provenance through a single parameter, *a*, the age at which trees transition to the adult stage. This toy model will help us better understand the results of more complex life cycles. Figure 2 shows how the number of surviving trees in a cohort with a given life history varies depending on the thermal optimum of the planted provenance. Our equations predict that, quite intuitively, the best provenance to plant has a higher thermal optimum ($T_{\mathrm{tot}}\left(\vec{a}\right)$ as in Equation 6), i.e., is more warm‐adapted, when climate change is faster or when rotation time is longer (as illustrated in Figure 2). The best provenance, however, does not vary with the cumulative thermal tolerance ($\omega_{\mathrm{tot}}\left(\vec{a}\right)$; compare panels a and b in Figure 2), depending only on the relative change in thermal tolerance across the lifespan as shown by Equation 5. Interestingly, even when planting the best provenance, the maximal number of surviving trees in the cohort (i.e., $s_{\mathrm{tot},\max}\left(\vec{a}\right)$ as in Equation 7) is less in scenarios where the climate changes within the course of tree life than in the absence of climate change (compare the coloured curves in Figure 2). Our model predicts that assisted gene flow can therefore, at best, only partly mitigate the negative consequences of a warming climate. The part of unavoidable loss in harvest increases with more rapid climate warming, longer time before harvest (compare full to dot‐dashed line in Figure 2) and when trees have a narrower climatic niche (i.e., smaller cumulative tolerance over their lifetime, compare panels a and b in Figure 2).

**FIGURE 3:**
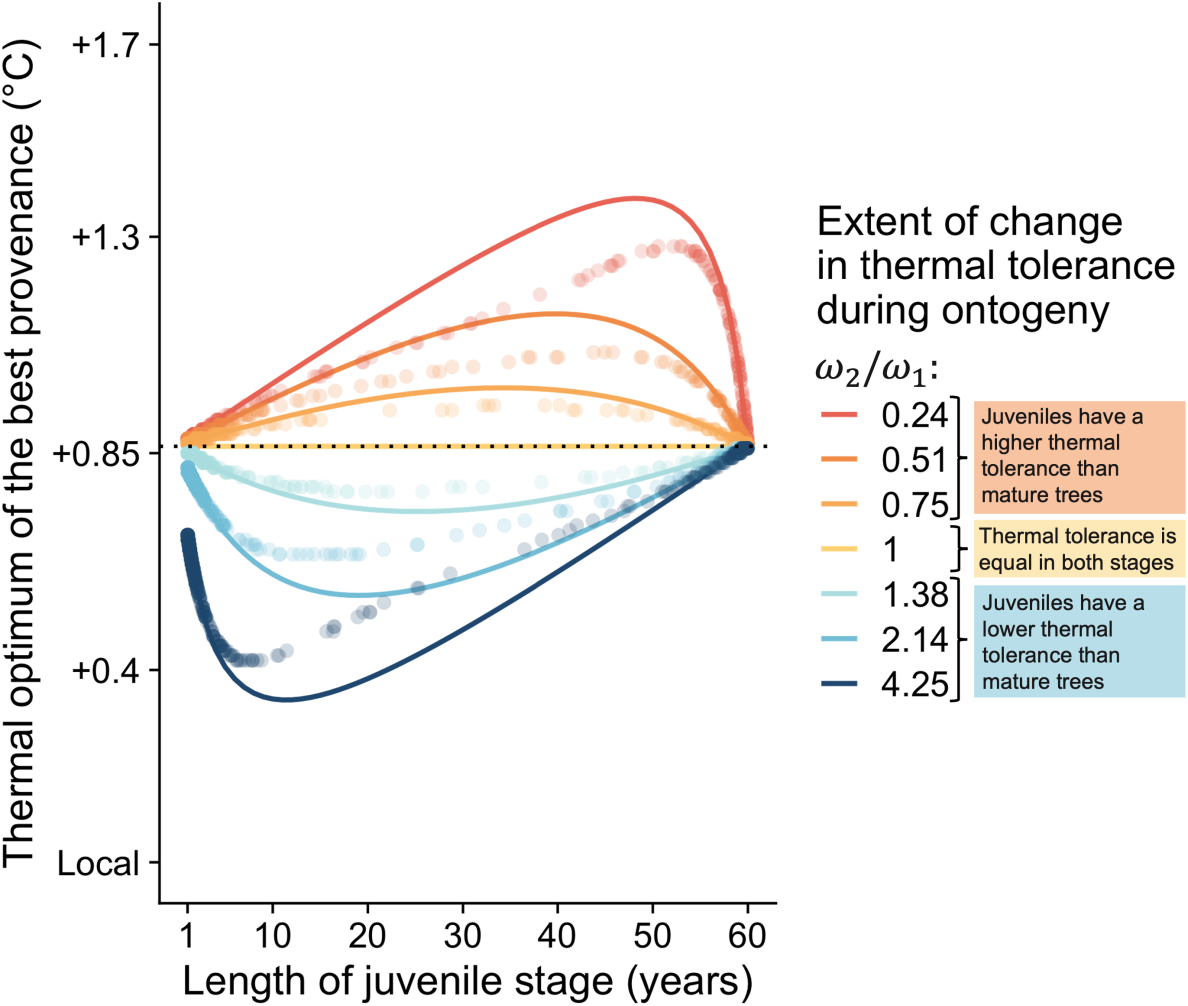
The best provenance is determined by variation in thermal tolerance across life stages (coloured curves), and the duration of the juvenile phase. The analytical results from a two‐stage model are illustrated with a climate warming scenario of +1.7°C in 60 years. The extent of the change in thermal tolerance during ontogeny is represented by the ratio between the thermal tolerance of mature trees and juvenile trees (*ω*_2_/*ω*_1_), shown with colours. Solid lines represent the thermal optimum of the best provenance using the age‐structured analysis, that is, assuming the whole cohort grows from juveniles to mature trees at age *a* (predicted using Equation 3). Dots show the thermal optimal provenance in a stage‐structured framework, i.e., when individuals stochastically can grow at different ages (predicted using Equation 8), for 500 sets of random values for *g*, *s*_1,max_ and *s*_2,max_ drawn from uniform distributions between 0.01 and 0.99, conditional upon survival. The x‐coordinate gives the average maturation age among individuals surviving to harvest for each set of life‐history parameters. The dotted horizontal line indicates the average temperature between planting and harvest.

Figure 3 shows how life history influences the best provenance for seeds. If thermal tolerance is equal in both stages $\left(\omega_{2}/\omega_{1}=1\right)$, the thermal optimum of the best provenance does not depend on the age of maturation from juvenile to mature tree $\left(a\right)$. When tolerance changes across the tree's life, the thermal optimum of the best provenance is, however, pulled towards the temperatures experienced in the least tolerant stage, with the extent of this pull determined by the duration of this stage (Figure 3). Time spent in the least tolerant stage furthermore has a non‐monotonic effect on the choice of the best provenance: when juveniles are less tolerant to temperature changes and the juvenile period is short, increasing the length of the juvenile period makes the choice of cooler‐adapted provenance more advantageous, but when this juvenile period is very long, it is best to select warmer‐adapted provenances. If time spent in either phase is very long (such that the trees experience most years in that stage), the thermal optimum of the best provenance is close to the average temperature experienced during the period. Differences in thermal tolerance across life stages matter most when the age of the transition between stages is intermediate, such that a significant part of life is spent in each stage.

The above line of reasoning applies when all individuals have the same life history and should work well to predict the thermal optimum of the best provenance in a stage‐structured population if there is little variation in life history among those that survive until harvest. In Figure 3, we compare the thermal optimum of the best provenance as a function of the mean age of maturation among survivors at harvest predicted in the stage‐structured model (dots) to analytical predictions for a fixed age of maturation between stages (curves). All previous qualitative conclusions about the thermal optimum of the best provenance, assuming a fixed life history, hold in the stage‐structured model, and the quantitative predictions assuming a constant life history lack precision only when the age of transition between stages is intermediate. In those cases, the mean age at transition hides a great deal of variation in life histories, with some individuals maturing early and others late. Stochastic realizations of each individual's life history then tend to lengthen one stage or the other, causing the optimum in the stage‐structured model to be a bit less sensitive to differences in thermal tolerance than the strictly age‐structured model (dots are closer to the case where $\omega_{1}/\omega_{2}=1$ than the lines in Figure 3).

We find that relaxing our assumption of a homogeneous cohort (i.e., increasing the phenotypic variance at the plantation site) is unlikely to change our predictions about the best provenance much (Equation S1b and Figure S1). In the scenario of a variable cohort, we observe that increasing the initial phenotypic variance decreases the expected number of survivors when planting the best provenance, but that higher phenotypic variance can minimize the loss of survivors when planting a subpar provenance, for example, if climate deviates strongly from our expectations (Equation S1b, Figure S2).

### 3.2 Simulation results

#### 3.2.1 Optimal provenance for 20 non‐tropical tree species in a warming climate

**FIGURE 4:**
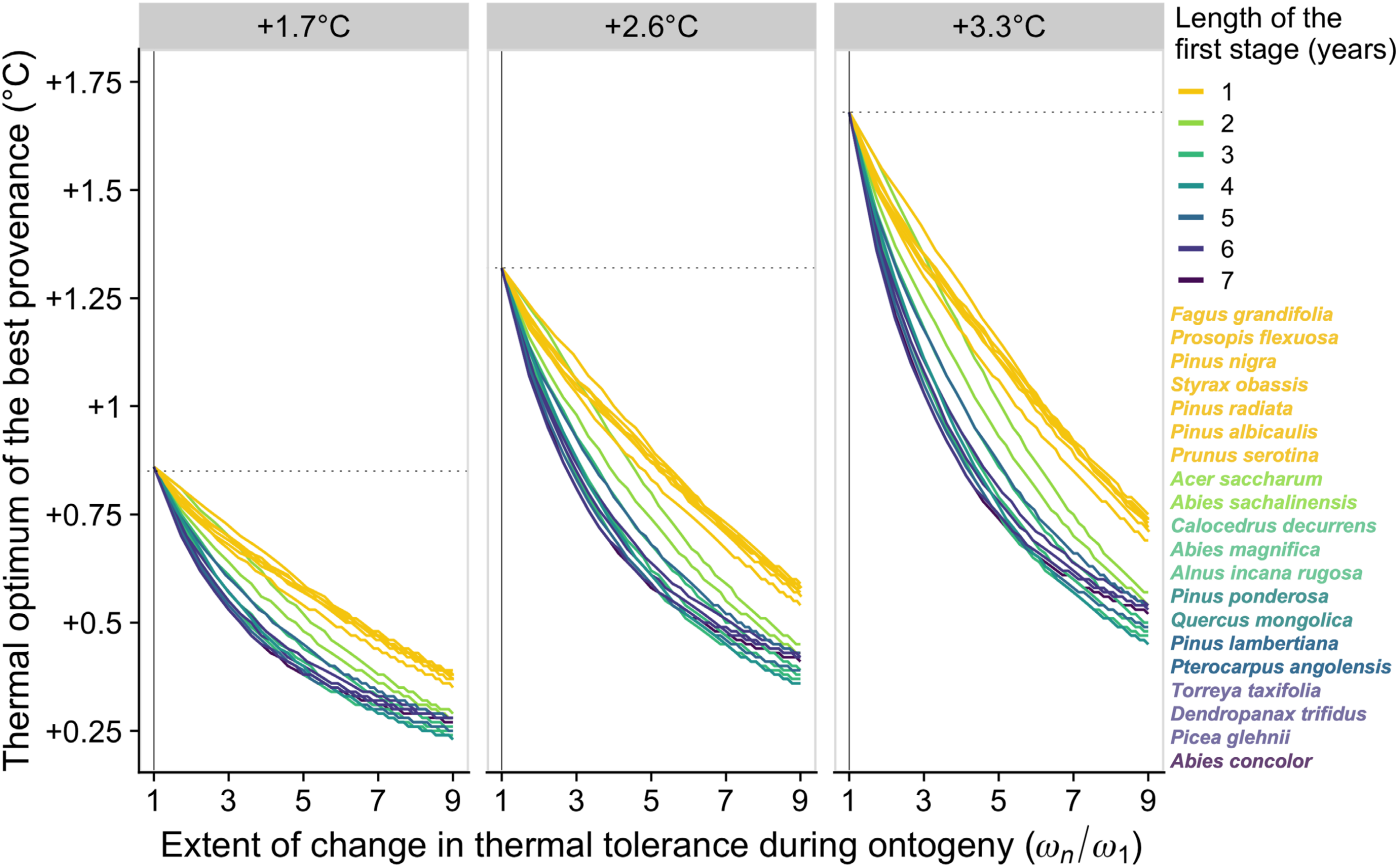
Variation in the best provenance of a plant is illustrated using the life‐history trajectories of 20 long‐lived tree species. The less tolerant younger trees are to changes in temperature compared to older trees (the larger *ω*_*n*_/*ω*_1_ along the *x*‐axis), the cooler the best provenance. The effect is shown for 3 warming scenarios (panels). Each curve represents a species and is coloured by the length of their first life stage (in transition matrices that consider seeds or seedlings, individuals spend only 1 year in this first stage). The vertical line indicates the case where tolerance is constant across all stages. For each warming scenario, the dotted horizontal lines correspond to the average temperature over the period.

In accordance with the analytical predictions, when the thermal tolerance is constant over the lifetime of the trees $\left(\omega_{n}/\omega_{1}=1\right)$, the thermal optimum of the best provenance matches the average temperature over the period and does not vary across species (but is warmer if temperatures rise faster) (Figure 4). When young trees are less tolerant to temperature changes than old trees $\left(\omega_{n}/\omega_{1}>1\right)$, the thermal optimum of the best provenance decreases, as predicted analytically, and becomes increasingly different among species depending on their life history (see Supplementary Material S6 for similar conclusions for species with longer rotation times). The optimal provenance for species that spend many years in the first and least tolerant stage is cooler and much nearer current temperatures (darker curves in Figure 4) than for species that mature earlier (lighter curves). The ranking of species can reverse if this first stage is both poorly tolerant to climate variation and prolonged, as young individuals will then be exposed to warmer temperatures. This pattern is consistent with the non‐monotonic effect of changing the age at the transition between stages shown in Figure 3. We, however, caution that stage‐specific temperature sensitivities are likely to differ between species and that empirical data on $\omega_{i}$ across the lifespan of trees is needed for species‐specific predictions.

#### 3.2.2 Comparing the performance of different seed sources

**FIGURE 5:**
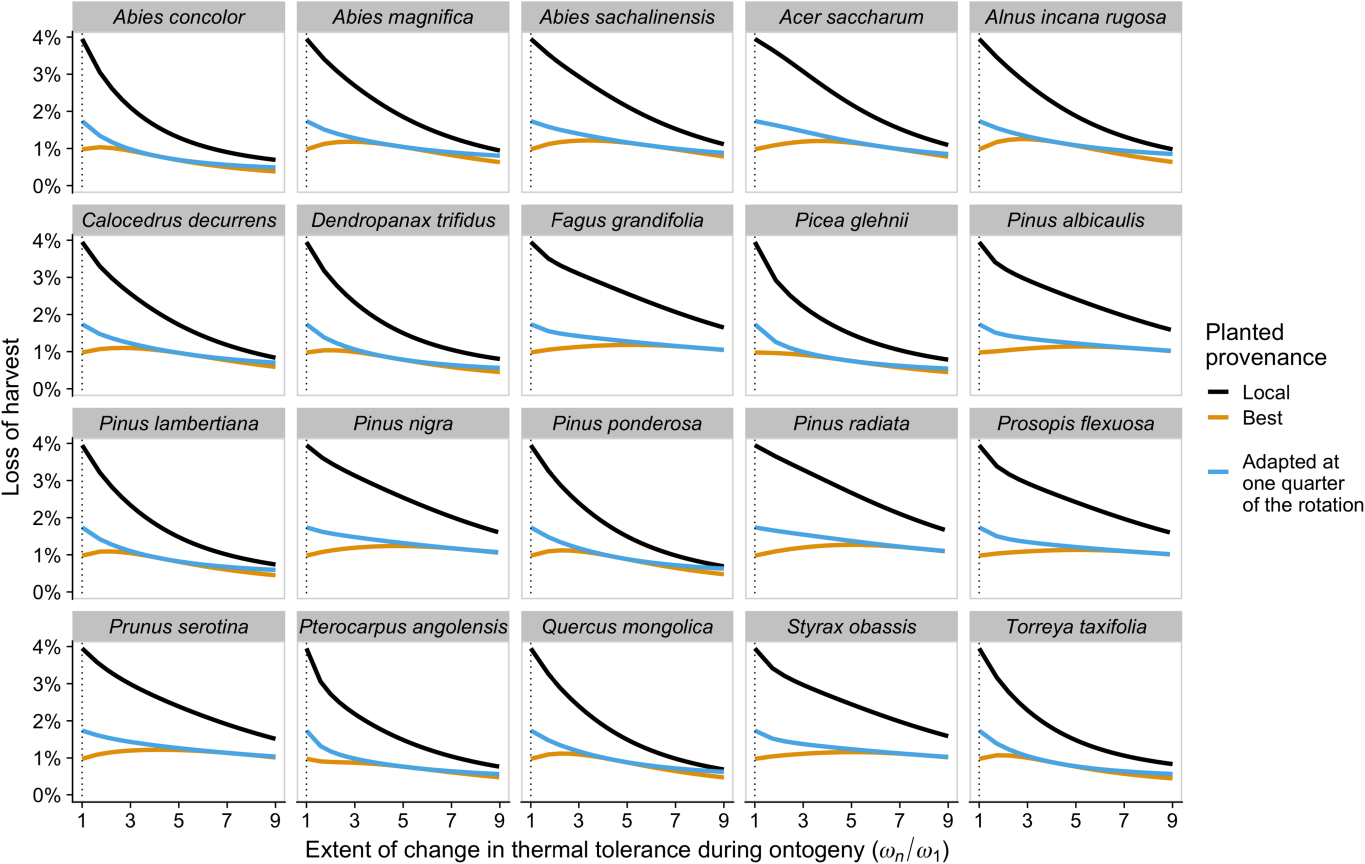
Large losses in harvest could be avoided for all species by planting the best provenance (yellow) or the provenance adapted to the temperature at one quarter of the rotation time (+0.43°C; blue) as opposed to planting the local provenance (black), but the benefit is reduced if young trees are less tolerant to changes in temperature as compared to older trees (*ω*_*n*_/*ω*_1_ increases) in an intermediate climate warming scenario of +1.7°C. The cumulative thermal tolerance is held fixed across the lifespan at *ω*_tot_($\vec{a}$) = 3.5°C. Harvest loss is measured against the performance of the local provenance in a scenario with no climate change. The vertical dotted line indicates the case where tolerance is constant across all stages.

We seek to assess the accuracy of using simple rules for provenance selection, considering that critical parameters, such as how thermal tolerance changes over a species lifespan, are often unknown. In our simulations, we consider three seed sources: (1) the “local” source, which we here assume to be adapted to the current climate with optimal temperature matching the expected temperature when the cohort is planted, (2) the model‐predicted best provenance and (3) a provenance adapted to the predicted temperature at one quarter of the rotation time (i.e., in the present scenario in 15 years), as suggested by O'Neill et al., 2017. We compare the predicted harvest loss when planting each seed source (Figure 5 and Supplementary material S7: Figure S7). For all species examined, our model predicts that the benefit of planting the best provenance (yellow) compared to the local provenance (black) is significant when all stages are assumed to be equally tolerant to temperature changes: using the best provenance instead of the local provenance then allows reducing loss from 4% to <1% in Figure 5, and these gains are considerably greater if climate change is faster and/or cumulative thermal tolerance is set to be smaller (Supplementary material S7: Figure S7). The benefits of assisted gene flow are, however, predicted to diminish when younger trees are much less tolerant than older trees $\left(\omega_{n}>>\omega_{1}\right)$, with the best provenance performing only slightly better than the local provenance (Figure 5 and Supplementary material S7: Figure S7). This happens because the best provenance is then cooler‐adapted and less different from the local provenance. Our model predicts that the recommendation from O'Neill et al., 2017 (blue) performs nearly as well as the best provenance (yellow) when juveniles are less tolerant to temperature changes than older trees, but not quite as well if all stages are equally tolerant (at most, the loss of harvest is 2% as opposed to the 1% for the best provenance in Figure 5).

#### 3.2.3 Climate fluctuations

**FIGURE 6:**
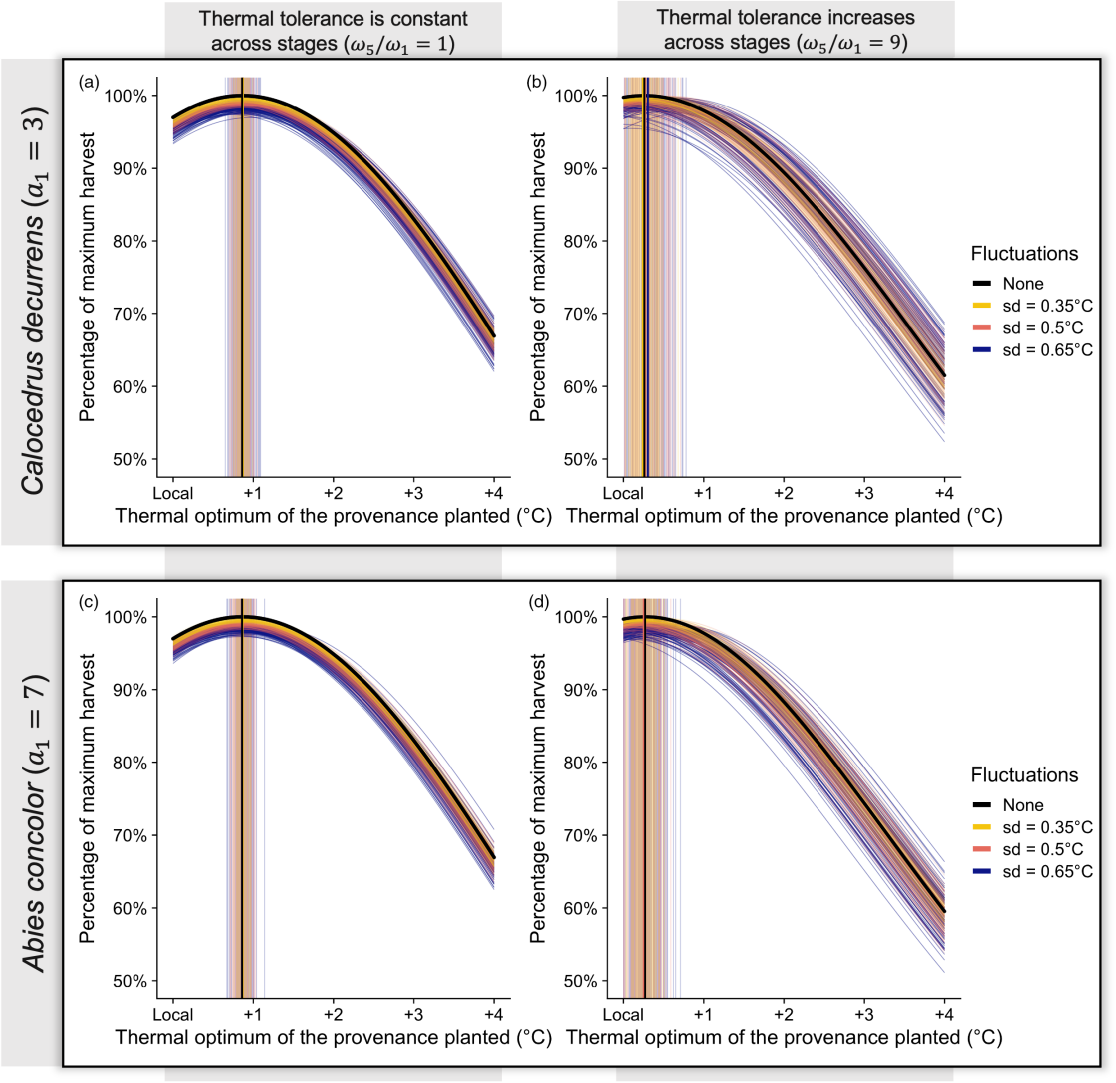
Random interannual fluctuations can lead to a reduced harvest for *Calocedrus decurrens* (panels a and b) and *Abies concolor* (panels c and d), but with increased variability when young trees are less tolerant to changes in temperature as opposed to older trees (*ω*_5_/*ω*_1_ = 9 for panels b and d vs. *ω*_5_/*ω*_1_ = 1 for panels a and c), and for *Calocedrus decurrens*, a species that remains in the first stage for a shorter time. Environmental temperature increases under the intermediate climate warming scenario of +1.7°C either linearly (black), with a variance given by the global average of locally observed values for interannual fluctuations (sd = 0.5°C; pink) or with a lower or higher variance (sd = 0.35°C; yellow, or 0.65°C; blue). Each scenario of climate warming with fluctuations is replicated 100 times with random draws of temperature around the warming trend. Each curve represents the harvest for each provenance relative to the harvest of the local provenance without climate change. The light vertical lines represent the thermal optimum of the best provenance for each run with fluctuations. The darker vertical lines represent the average of such thermal optima. The cumulative thermal tolerance across the lifespan is held fixed at *ω*_tot_($\vec{a}$) = 3.5°C.

We assess the robustness of the previous predictions by letting the annual temperature fluctuate from year to year around a warming trend. Fluctuations in the average annual temperature result in a reduction in harvest on average (Figure 6). We, however, find that the provenance that performs best on average is not different from the one estimated without fluctuations (Figure 6). There is nonetheless a lot of variability among runs (and thus uncertainty) in the thermal optimum of the best provenance with temperature fluctuations, indicating that the realized climate trajectory may randomly have been warmer or colder than expected, which would favour a warmer or cooler provenance, respectively. This variance in best provenance across realized climate trajectories is much larger when stages have different thermal tolerances (Figure 6b,d) and when the least tolerant stage lasts only for a few years (compare Figure 6b,d): when the first stage is poorly tolerant to changes in temperature, the best provenance depends primarily on the temperatures experienced randomly during the first few years of life: individuals must be well adapted to the temperatures in their first years to survive. In particular, if those early years are unusually cold, the thermal optimum of the best provenance to plant may even be cooler than the local provenance. The variability in harvest associated with interannual fluctuations is, however, smaller if diversified provenances are planted (Figure S2).

## 4 DISCUSSION

### 4.1 The best seed source for assisted gene flow in long‐lived species depends on how tolerance to climate changes throughout life

Several authors have suggested that assisted gene flow could mitigate the negative effects of climate change (e.g., Browne et al., 2019; Milesi et al., 2019). Recent modelling, however, suggests that the short‐term conservation benefits of assisted gene flow may often be modest in large populations unless pre‐adapted genetic variants with large effects are introduced at large frequencies (Grummer et al., 2022). This makes the identification of pre‐adapted sources of seeds particularly critical for the success of assisted gene flow in forest trees. The choice is indeed complicated by a trade‐off in long‐lived species, where adaptation to a warmer climate at the adult stage may come at the cost of poor adaptation to cooler climates while still juvenile. We have modelled the survival of a single cohort of trees in a changing climate and showed that, to maximize the number of surviving trees, the choice of the best provenance critically depends on differences between life stages in their tolerance to climate. When thermal tolerance is constant over the lifespan of a tree, the best provenance is the one whose thermal optimum corresponds to the average temperature observed over this period, which increases with the rate of climate warming before harvest. When there is stage‐specific thermal tolerance, the best provenance is pulled towards the temperature experienced in the least tolerant stage, weighted by how many years are spent in that stage. The thermal optimum of the best provenance is cooler than the average temperature across the period when young trees are less tolerant to changes in temperature and warmer when older trees are less tolerant. This effect of time spent in the least tolerant stage is complicated because the longer individuals stay in this stage, the more variable temperatures they experience while in that stage. We showed the predictions of this simple model to be quite robust to variation in the tree life cycle and the number of stages, the presence of variation in the climatic niche within the provenance and the stochastic fluctuations of climate around the warming trend. While we have focused on temperature for the sake of illustration, the same conclusion would apply for any climatic variable or combination of climatic variables affecting tree growth and survival (e.g., precipitation).

### 4.2 We lack information on critical parameters to determine the best seed source

Our model shows that changes in tolerance during the life of the species are particularly relevant in determining the best provenance for assisted gene flow. Yet, quantitatively estimating these changes in different species throughout the life of a tree is not trivial. There is a need for further empirical estimates of how tolerance to climatic variables varies with tree ontogeny and affects the growth and survival of trees at different life stages. Some studies have attempted to answer these questions by looking at changes in the stage distribution of trees across space along climatic gradients (e.g., Bell et al., 2014; Lenoir et al., 2010; McLaughlin & Zavaleta, 2012), but lags in responses to climate change confound the interpretation of these patterns (Heiland et al., 2022). Long‐term provenance trials, where different seed sources are planted on multiple sites, are invaluable for assessing the risks and benefits of assisted gene flow. While there are older trials for some common species (e.g., Carter, 1996; Rehfeldt et al., 1999; Schmidtling, 1994; St. Clair et al., 2022), establishing new common gardens, with responses to climate changes in mind (such as Du et al., 2019; Kueppers et al., 2017; Munier et al., 2010), should be a priority. Another approach is the analysis of tree rings in regards to past climate change. Several such studies reveal that trees of different age and size vary in their growth response to past climatic variation (see Au et al., 2022; Carrer & Urbinati, 2004; Depardieu et al., 2020; Housset et al., 2018; Latreille et al., 2017; Marquis et al., 2020; Mašek et al., 2021; Pompa‐Garcıa & Hadad, 2016). Unless the entire historical population can be sampled (including dead trees), studying only those individuals who survived past climatic fluctuations may, however, lead to an overestimation of tolerance in older age classes due to survivor bias (Duchesne et al., 2019, similar to the slow‐grower survivorship bias reported in Brienen et al., 2012).

While younger life stages are generally thought to be less tolerant to climatic stress than larger trees (e.g., Black & Bliss, 1980; Kueppers et al., 2017; Munier et al., 2010; Pompa‐Garcıa & Hadad, 2016, though see Du et al., 2019 for an exception), several considerations may make our predictions about the best provenance more complex. First, younger and older stages may be sensitive to different climatic factors, rather than overall less or more tolerant to any stress (Mašek et al., 2021). Competition within and between species is a strong determinant of survival, especially in younger trees (Kunstler et al., 2021). Climate‐associated mortality in young stages may be balanced by reduced density‐dependent mortality. The effects of competition may therefore make seedling survival less sensitive to climatic conditions, favouring the choice of warmer‐adapted provenances for plantations. The exact practices to implement assisted gene flow could also affect our predictions: for instance, greenhouse or nursery cultivation of seedlings before transplantation makes their survival less affected by local temperature than trees regenerating from seed in colder natural environments.

Finally, even if our model correctly predicts the thermal optimum of the best provenance, information about the thermal optimum is not available for many potential seed sources. Given that climate change has already increased temperatures by an average of around one degree C globally (IPCC, 2021), current temperatures may not match the thermal adaptation of local sources that are adapted to pre‐Anthropocene temperatures (as, for instance, *Quercus lobata*, see Browne et al., 2019). Predictions of climate adaptation based on genomic data and genetic–environment associations are increasingly considered as an alternative to the assumption of systematic local adaptation, but these methods are also in need of further validation (Capblancq et al., 2020). Our model also assumes that the temperature maximizing the survival of an individual is the same for all its life, which is not necessarily the case if juveniles and adults have different climatic preferences. While it is straightforward to modify our model to include this possibility, we critically lack information about differences in thermal optimum between adults and juveniles in most species.

### 4.3 Uncertainties remain high about the costs/benefits ratio of assisted gene flow

Many uncertainties must be considered when discussing the balance of costs and benefits associated with shifting genotypes in response to or in anticipation of climate change (Srivastava et al., 2021).

The first level of uncertainty relates to the identification of the provenance that will perform best in a given climate scenario. Our model predicts how much tree mortality could be avoided by planting sources better adapted to the future climate than the local source. Interestingly, we found that these losses can be reduced even when information to identify the best provenance is not available by using a simple rule of thumb, such as planting provenances well adapted to the expected climate at one quarter of the rotation period. Using the best provenance also does not avoid all loss in a changing climate because of the trade‐off between early and late adaptation, described as the cost of within‐generation selection in Cotto and Chevin (2020). Our model predicts that planting warmer provenances in a warming environment can reduce tree mortality in a changing climate, but the benefits are only modest if climate change is not very fast, climatic tolerance is wide and young trees are much less tolerant to climate than old ones. The costs of translocation could therefore outweigh the advantages when early life stages are less climate‐tolerant than later stages. Indeed, populations are not only climate‐adapted but also locally adapted to other factors such as photoperiod, pollinators and pests (see Giencke et al., 2018 for an example of phenological mismatch and Wadgymar et al., 2022 for a review on local adaptation in plants). Translocated warmer provenances might, therefore, underperform compared to local provenances, even if they are better matched to the future climate. The benefits of assisted gene flow, however, increase if the local provenance is already maladapted to the current climate because of past climate warming: a warmer‐adapted seed source may then increase the survival of both early and late stages.

Our model also reveals a second level of uncertainty, the fundamental uncertainty associated with interannual variation in climate and the prediction of future climate. Differences in climatic tolerance between stages make predicting the best provenance of seeds with interannual variation more difficult than when differences between stages are ignored. On average, the best provenance that maximizes survival is not affected by these fluctuations, but in any realization of a sequence of climatic years, the best provenance may differ greatly from this average. High fluctuations around the expected performance of a provenance will be especially pronounced when there are strong differences in climatic tolerance between stages and when sensitive stages are short and so do not average across many years of climate variation.

## 5 CONCLUSIONS

This theoretical study helps answer questions about assisted gene flow in long‐lived species. Our model suggests that planting seeds from warmer sites in a warming environment has the potential to mitigate mortality losses for a tree cohort, even under an optimistic climate change scenario. Importantly, the length of time spent in each stage and the tolerance of that stage to the expected climate can also be used to identify which stages are expected to experience high mortality, which may help managers allocate resources across the life cycle (e.g., by thinning to reduce competition or spraying for pests during that stage). A great deal of uncertainty, however, remains about optimal seed sourcing, particularly because variation in tolerance to different climatic variables as a function of tree age or stage is not well known. This deserves to be investigated further empirically, alongside more extensive modelling of assisted gene flow, simulating mixing seed sources and alternative regeneration approaches, to better quantify the risks and benefits associated with assisted gene flow practices.

## CONFLICT OF INTEREST STATEMENT

The authors declare no conflicts of interest.

## Supporting information


Appendix S1.

Data S1.

Code S1.

Code S2.

Code S3.

## Data Availability

The data that support the findings of this study is openly available in public databases (COMPADRE – Plant Matrix Database, IPCC WGI Interactive Atlas).
