# Supplementary material for: Planting long‐lived trees in a warming climate: Theory shows the importance of stage‐dependent climatic tolerance
Source: Evol Appl. 2024 Jun 17;17(6):e13711. doi: 10.1111/eva.13711 (PMC11183180; doi:10.1111/eva.13711)
Supplement: Supplementary file 1 — Appendix S1. [file EVA-17-e13711-s001.pdf]

Supplementary material

S1. Increasing phenotypic variance at the plantation site

We extend the two-stage framework developed in the main text, assuming here that phenotypes are normally distributed with mean phenotype $\bar{\theta}$ and phenotypic variance P_0. We apply the age-structured result of Cotto and Chevin (2020) for multiple rounds of Gaussian selection on a single cohort (their equation 9) to write the expected number of mature trees at harvest time if all surviving trees had grown from juveniles to mature trees at age a, as:

$$E[N_H(\bar{\theta})|a] = N_0\, s_{\text{tot,max}}(a)\sqrt{\frac{\omega^2_{\text{tot}}(a)}{P_0 + \omega^2_{\text{tot}}(a)}}\exp\left(-\frac{(\bar{\theta} - T_{\text{tot}}(a))^2}{\omega^2_{\text{tot}}(a) + P_0}\right) \tag{S1a}$$

Considering all possible life histories until harvest, the remaining pool of individuals is formed by the sum of the cohorts of individuals making a transition at the same age a:

$$E[N_H] = g\, N_0 \sum_{a=1}^{H} (1-g)^{a-1} s_{\text{tot,max}}(a)\sqrt{\frac{\omega^2_{\text{tot}}(a)}{P_0 + \omega^2_{\text{tot}}(a)}}\exp\left(-\frac{(\bar{\theta} - T_{\text{tot}}(a))^2}{\omega^2_{\text{tot}}(a) + P_0}\right) \tag{S1b}$$

The range of optimal annual temperatures maximizing survival over 20 years among different provenances of *Pinus contorta* was found to span over 7.6°C (Rehfeldt et al. 1999). We set the phenotypic variance (P_0) in the plantation to 2°C^2 for a "moderate" variance scenario and to 6°C^2 for a "high" variance scenario, mimicking a situation where different provenances would be planted together in the cohort.

We find that increasing phenotypic variance at the planting site does not affect our prediction for the best provenance, but it does reduce the expected number of survivors when the best provenance is planted (Suppl. Fig. 1). This makes sense as fewer individuals will have the optimal phenotype in the initial cohort. When the planted provenance is very different from the best provenance, phenotypic variance among the planted trees conversely increases the harvest, because the cohort is more likely to contain some untypical individuals with a phenotype closer to the optimum.

We next investigate if these conclusions hold in less predictable environments when the temperature fluctuates around a warming trend. In agreement with Supp. Fig. 1, we find that increasing phenotypic variance tends to decrease the average harvest (Supp. Fig. 2), but also decreases variance in harvest between runs and avoids very large loss when maladapted provenances are planted.

Distributed under a Creative Commons Attribution — 4.0 International licence

Effect of increasing phenotypic variance

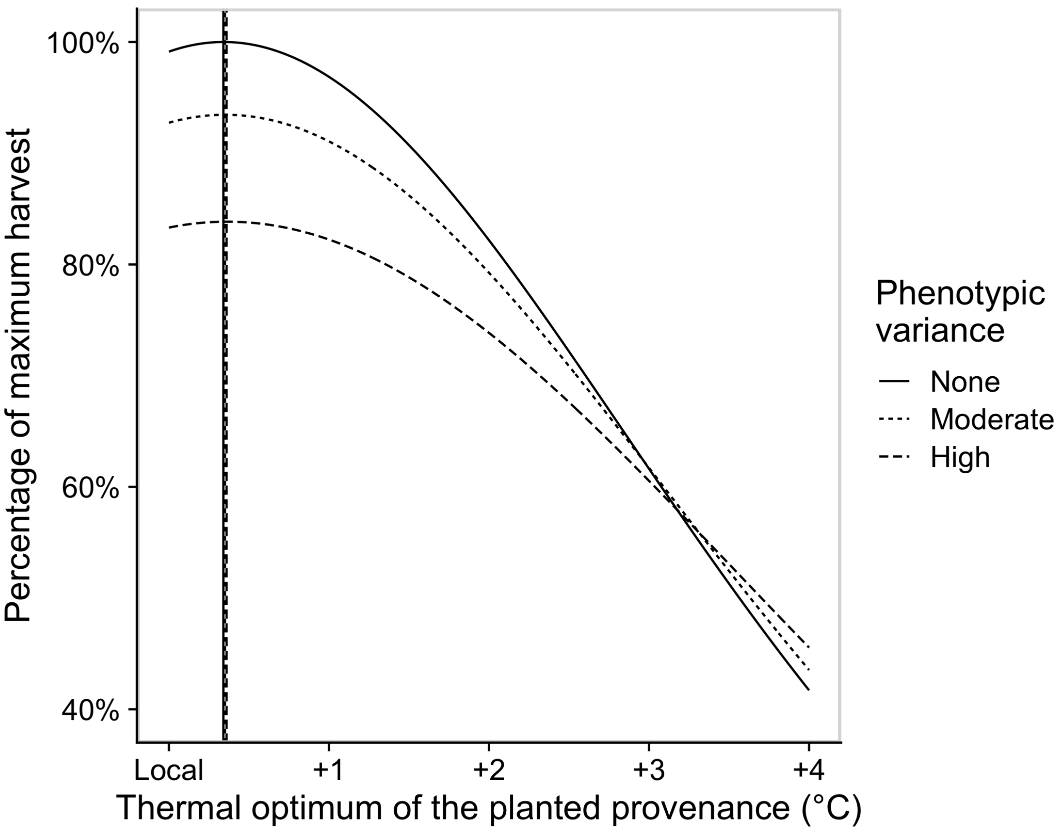

Suppl. Fig. 1: Increasing phenotypic variance at the planting site has no effect on the prediction of the thermal optimum of the best provenance (vertical lines), but it does reduce the harvest when the best provenance is planted. When the thermal optimum of the planted provenance planted is very far from that of the best provenance, however, phenotypic variance increases the expected harvest. This result is depicted for a climate warming scenario of +1.7°C in 60 years and using parameters: $s_1 = 0.61$, $s_2 = 0.98$ and $g = 0.012$. Three scenarios of phenotypic variance are considered ("None", $P_0 = 0°\text{C}^2$, solid line), moderate phenotypic variance ("Moderate", $P_0 = 2°\text{C}^2$, short dash) or high phenotypic variance ("High", $P_0 = 6°\text{C}^2$, long dash). Each curve represents the percentage of maximum harvest produced by each provenance in each scenario of phenotypic variance. The curves were produced with the two-stage stage-structured model (equation S1b). The cumulative thermal tolerance across the life span was held fixed at $\omega_{\text{tot}}(\vec{a}) = 3.5°\text{C}$ and the extent of change in thermal tolerance across the lifespan was set to $\omega_2/\omega_1 = 46$.

Distributed under a Creative Commons Attribution — 4.0 International licence

Effect of increasing phenotypic variance when the climate fluctuates

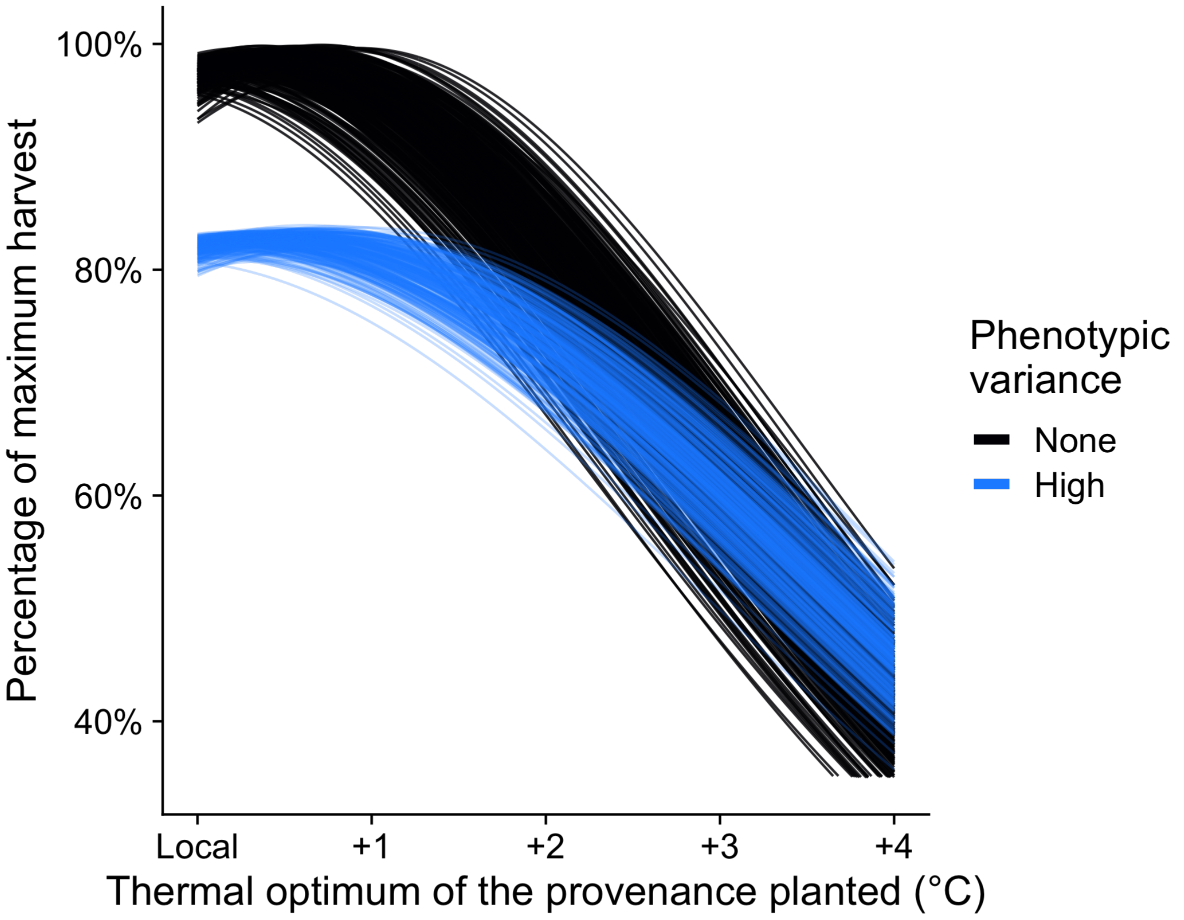

Suppl. Fig. 2: Increasing phenotypic variance (blue lines, $P_0 = 6°C$) under random inter-annual fluctuations in mean annual temperature generally results in lower harvest than in the case without phenotypic variance (black lines, $P_0 = 0°C$) but phenotypic variance limits the loss in the case where the provenance planted is very different from the best provenance for a climate warming scenario of +1.7°C in 60 years and using parameters: $s_1 = 0.61$, $s_2 = 0.98$ and $g = 0.012$. Environmental temperature increases with standard deviation around the trend of sd $= 0.65°C$. Each scenario was replicated 300 times. Each line represents the percentage of maximum harvest produced by each provenance in each scenario of phenotypic variance and for each random climatic trajectory. Cumulative thermal tolerance across the life span was held fixed at $\omega_{tot}(\vec{a}) = 3.5°C$. Thermal tolerance across the lifespan was set to $\omega_2/\omega_1 = 46$. The curves were produced with the two-stage stage-structured model (equation S1b).

Distributed under a Creative Commons Attribution — 4.0 International licence

S2. Both survival and growth are sensitive to temperature

If both the survival and growth rates depend on temperature, we define, as before, the survival rate in stage i (s_i), as a Gaussian function of the thermal optimum of the provenance θ_s and the current temperature T_t in year t, with $s_{i,\max}$ the maximal survival rate in stage i and $\omega_{s,i}$ the thermal tolerance for survival in stage i:

$$s_i(\theta_s, T_t) = s_{i,\max} \exp\left(-\frac{(\theta_s - T_t)^2}{2\,\omega_{s,i}^2}\right) \tag{S2a}$$

In this case, the growth rate in stage i (g_i) is also expressed as a Gaussian function of the thermal optimum of the provenance θ_g and the current temperature T_t in year t, with $g_{i,\max}$ the maximal growth rate in stage i and $\omega_{g,i}$ the thermal tolerance for growth in stage i:

$$g_i(\theta_g, T_t) = g_{i,\max} \exp\left(-\frac{(\theta_g - T_t)^2}{2\,\omega_{g,i}^2}\right) \tag{S2b}$$

We then apply the same framework as the case where only survival is dependent on temperature: each year, individuals can either remain in their current stage if they survive and do not grow sufficiently to transition (with probability $s_i(\theta_s, T_t) \times (1 - g_i(\theta_g, T_t))$), die (with probability $1 - s_i(\theta_s, T_t)$), or transition to the next stage if they survive and grow (with probability $s_i(\theta_s, T_t) \times g_i(\theta_g, T_t)$).

We focus on the effect of having both survival and growth dependent on temperature on the optimal provenance, and for simplicity assume that survival and growth have the same thermal optimum ($\theta_s = \theta_g$) and the same thermal tolerance ($\omega_{s,i} = \omega_{g,i}$). Note that, in this case, the effect on individuals of distance from the optimum is even more important, as it reduces both survival and growth. We therefore expect a lower number of survivors at harvest time.

For this analysis, we exclude species that spend only one year in the first stage. The definition of the first stage in these matrices is "seed" (see Supplementary material S4), with a probability of moving to the next stage set to 1. Making this transition probability dependent on temperature would make the duration of this seed stage longer than a year, which would be biologically implausible for these species.

Distributed under a Creative Commons Attribution — 4.0 International licence

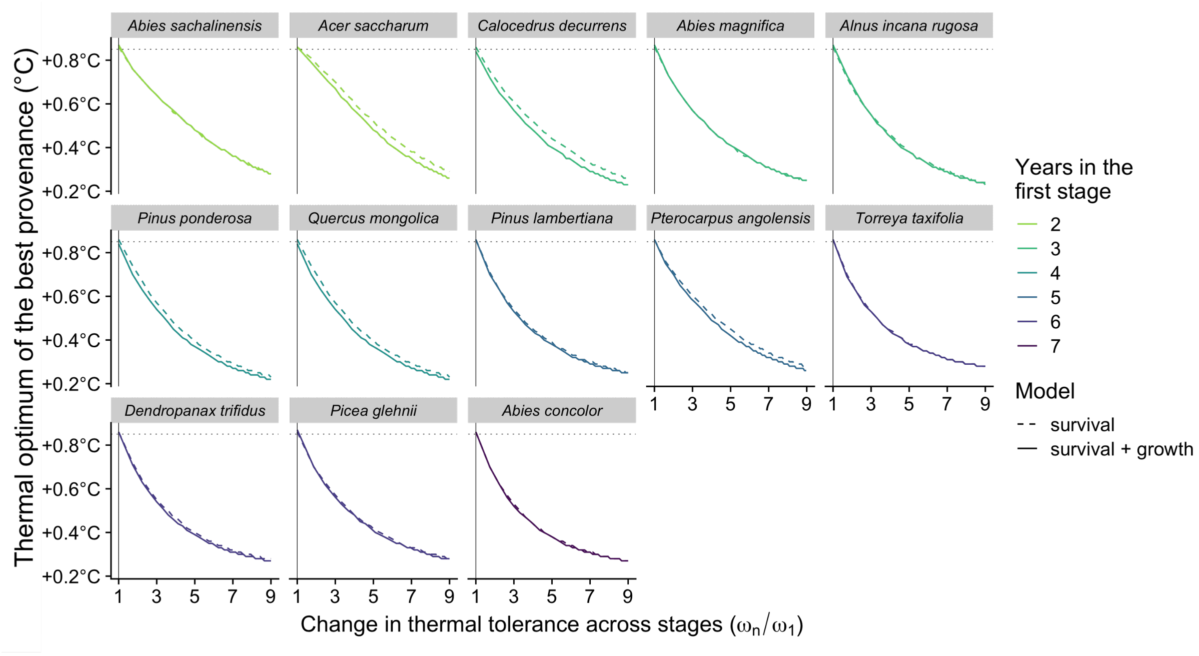

Suppl. Fig. 3: Comparison of model predictions for the thermal optimum of the best provenance as a function of changes in thermal tolerance across stages (ω_n/ω_1) when only survival is dependent on temperature (dashed lines) and when survival and growth are both dependent on temperature (solid lines) for thirteen tree species (panels) in the intermediate climate warming scenario of +1.7°C. Curves are coloured according to the length the first life stage of each species. In the case where survival and growth are both dependent on temperature, both share the same thermal optimum and thermal tolerance. The vertical line indicates the case where tolerance is constant across all stages. The dotted horizontal line corresponds to the mean temperature over the period. The total thermal tolerance across the life span is held fixed at $\omega_{\text{tot}}(\vec{a}) = 3.5$°C.

We find when both survival and growth depend on temperature, the thermal optimum of the best provenance decreases when young trees are less tolerant to temperature changes than old trees, matching the relationship described when only survival depends on temperature ($\omega_n/\omega_1 > 1$; Suppl. Fig. 3).In these examples, our predictions about the best provenance are also very close quantitatively to predictions when climate change only affects survival.

S3. Comparing analytical predictions with simulations

We compared analytical prediction given by equation 8 and the numerical simulations for a two-stage life cycle for which we varied the optimal temperature of the provenance θ between

Distributed under a Creative Commons Attribution — 4.0 International licence

the local temperature in the planting site to the local temperature +7°C, in increments of 0.01 degrees. Each simulation iterated the model for $H = 60$ years and computed the number of individuals that have reached the last stage at harvest. We found both model outputs to be identical (Suppl. Fig. 4).

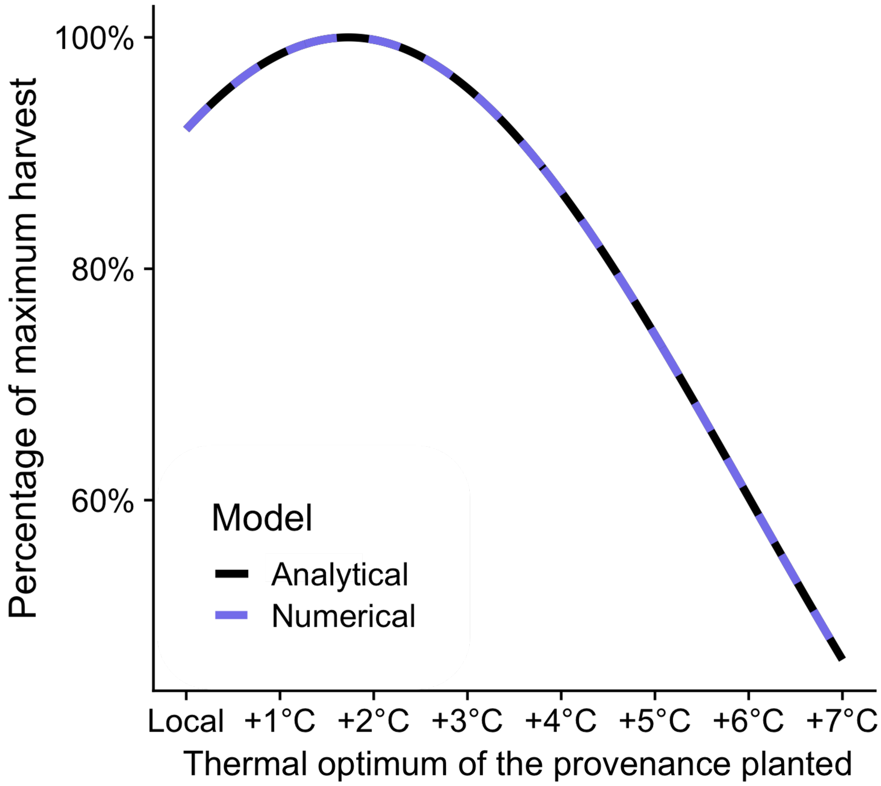

Suppl. Fig. 4: Comparison between analytical predictions (black) and numerical simulations (purple). The black curve was produced by the stage-structured analytical prediction (equation 8) and the purple curve by numerical simulations. Harvest includes all surviving individuals, regardless of their stage. The transition probabilities between stages were calculated with parameters: $s_1 = 0.61$, $s_2 = 0.98$ and $g = 0.012$. Simulations were carried out under the intermediate climate warming scenario of +1.7°C, and the harvest period was set at $H = 60$. Thermal tolerance was held constant across the lifespan ($\omega_2/\omega_1 = 1$) with the total thermal tolerance fixed at $\omega_{\text{tot}}(\vec{a}) = 3.5$°C.

S4. Details about the demographic matrices gathered

The transition matrices gathered are quite variable in the way that they categorize life stages, both in the definition and number of stages. For example, the first stage for the matrix of white fir (*Abies concolor*) contains individuals below 5cm in diameter at breast height and the second

stage individuals between 5 to 10cm DBH, while the matrix for tortuous mesquite (*Proposis flexuosa*) defines the first stage as "seedling", and the second stage as saplings with a diameter at ground level below 1 cm. Some of the variability stems from real biological differences (*e.g.*, with a maximum height ranging from 10 meters for tortuous mesquite to 75 meters for white fir), but some differences may be more arbitrary. In particular, some transition matrices include a transient seed stage before germination, while others start with seedlings (see Data S1).

S5. Expected warming at the geographic coordinates of the studied tree species

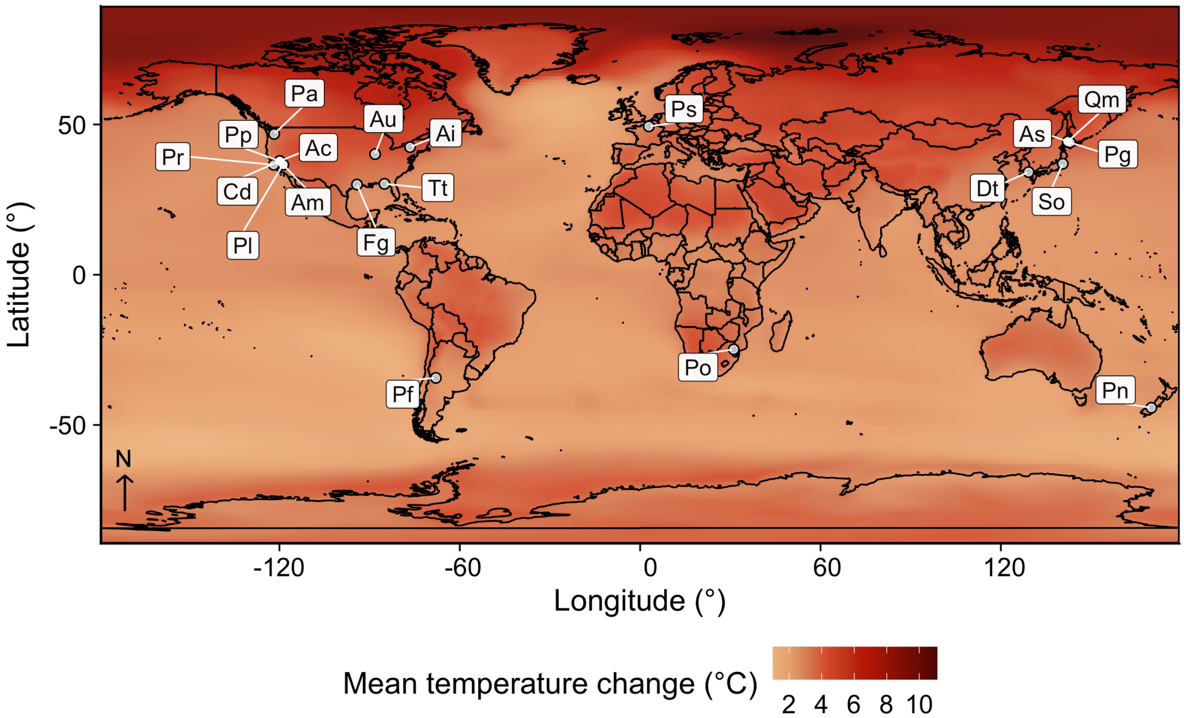

Suppl. Fig. 5: Geographic location of investigated tree species in our projected intermediate global climate warming of +1.7°C. The dots represent the geographic coordinates at the location of the tree population matrix estimation. Tree species are *Abies concolor* (Ac), *Abies magnifica* (Am), *Abies sachalinensis* (As), *Acer saccharum* (Au), *Alnus incana* (Ai), *Calocedrus decurrens* (Cd), *Dendropanax trifidus*(Dt), *Fagus grandifolia* (Fg), *Picea glehnii* (Pg), *Pinus albicaulis* (Pa), *Pinus lambertiana* (Pl), *Pinus nigra* (Pn), *Pinus ponderosa* (Pp), *Pinus radiata* (Pr), *Prosopis*

Distributed under a Creative Commons Attribution — 4.0 International licence

flexuosa (Pf), *Prunus serotina* (Ps), *Pterocarpus angolensis* (Po), *Quercus mongolica* (Qm), *Styrax obassis* (So) and *Torreya taxifolia* (Tt). The estimated mean temperature change is from the IPCC CMIP6 - SSP2-4.5 climate warming scenario projected for a long term period 2081-2100 (rel. to 1995-2014) (data can be downloaded at https://interactive-atlas.ipcc.ch).

S6. Longer rotation time

To test the effect of a longer harvest time ($H = 100$ years), we use three matrices of species that reach the last stage after 60 years and before 100 years. Details about the species matrices used can be found in Supporting Information (Data S1).

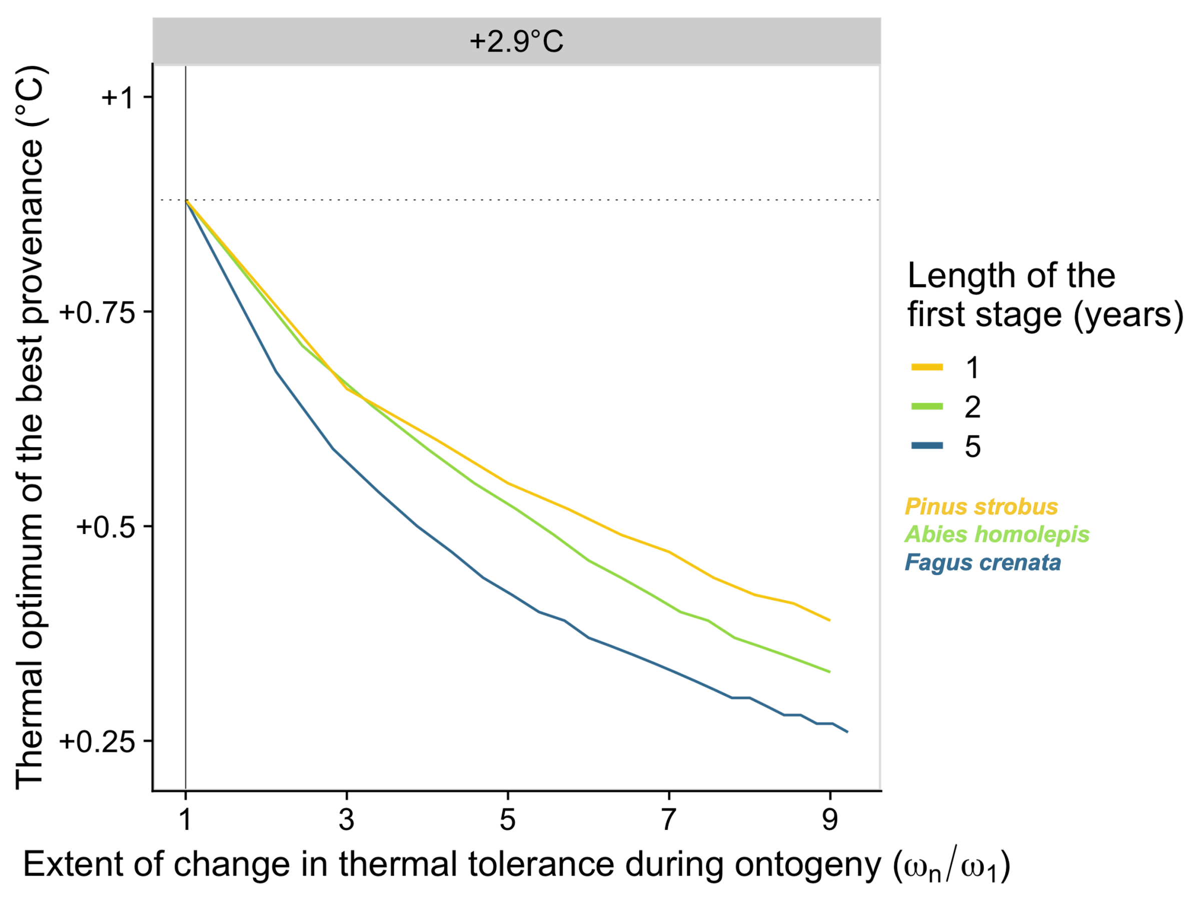

Suppl. Fig. 6: Variation in the best provenance to plant illustrated using the life history trajectories for three species that mature slower. The less tolerant younger trees are to changes in temperature compared to older trees (the larger ω_n/ω_1 along the x-axis), the cooler the best provenance. The effect is shown for the intermediate warming scenario. For a rotation time of 100 years, the intermediate warming scenario (of 1.7°C in 60 years) gives a warming of 2.9°C

in 100 years. Each curve represents a species and is coloured by the length of their first life stage. The vertical line indicates the case where tolerance is constant across all stages. For each warming scenario, the dotted horizontal lines correspond to the average temperature over the period.

We find that our main qualitative predictions also hold for species that mature slower with a longer rotation time ($H = 100$). When the thermal tolerance is constant over the lifetime of the trees ($\omega_i/\omega_1 = 1$), the thermal optimum of the best provenance matches the average temperature over the period and does not vary across species (Suppl. Fig. 6). When young trees are less tolerant to temperature changes than old trees ($\omega_i/\omega_1 > 1$), the thermal optimum of the best provenance decreases and becomes increasingly different among species depending on their life history. The optimal provenance for species that spend many years in the first and least tolerant stage is cooler and much nearer current temperatures (darker curves) than for species that mature earlier (lighter curves).

S7. Comparing the performance of different seed sources under multiple climate warming scenarios and for different values of the cumulative thermal tolerance ($\omega_{\mathrm{tot}}(\vec{a})$)

A: Climate warming = +1.7°C and $\omega_{\mathrm{tot}}(\vec{a}) = 2$°C

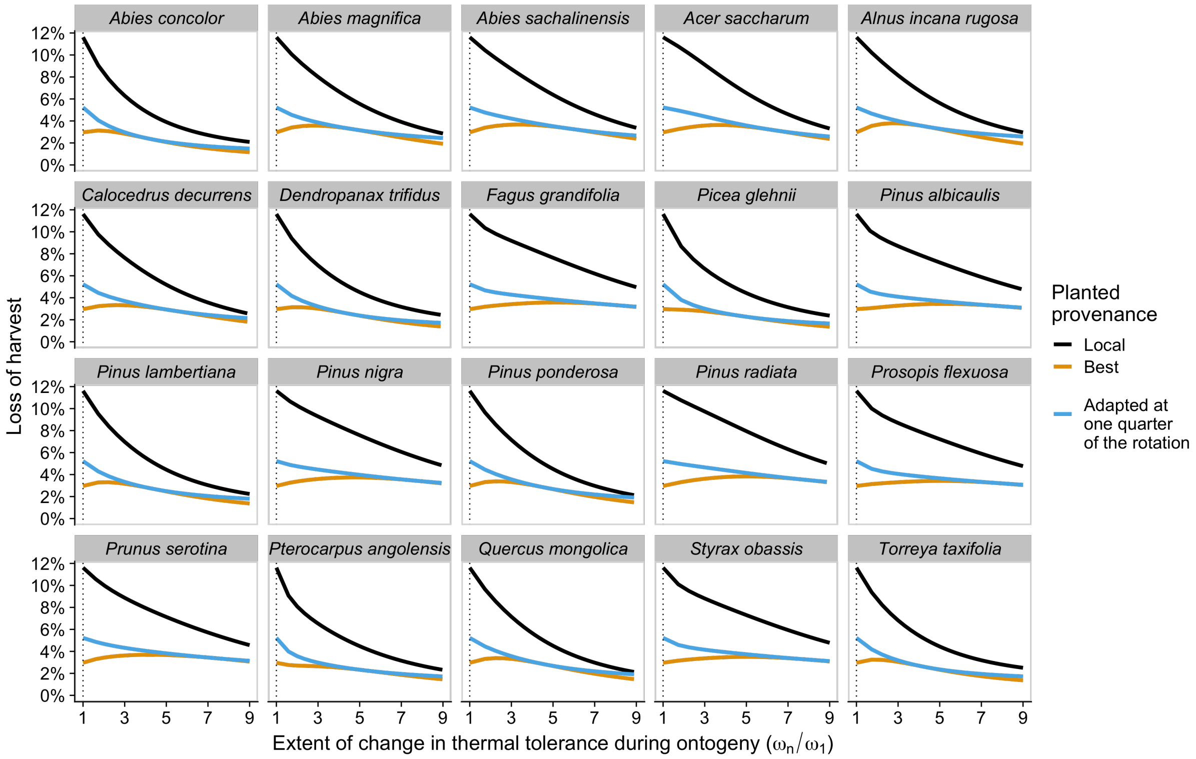

B: Climate warming = +1.7°C and $\omega_{\text{tot}}(\vec{a}) = 5$°C

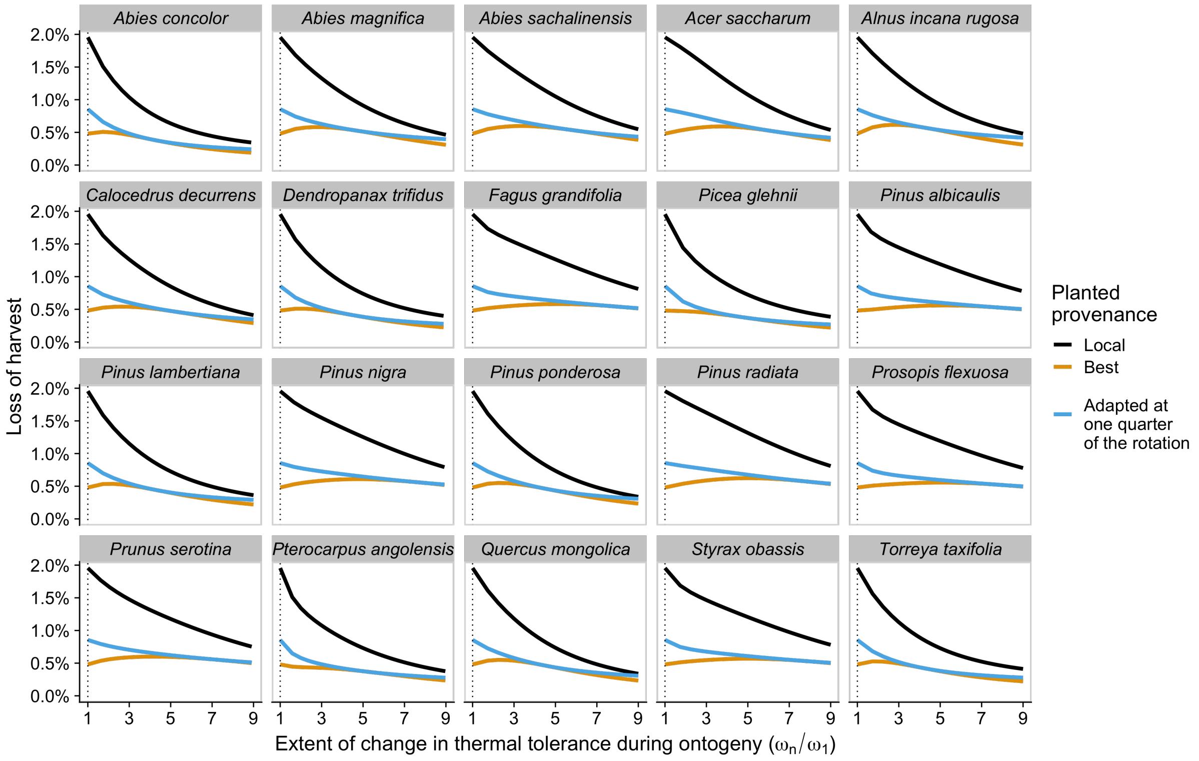

C: Climate warming = +2.6°C and $\omega_{\text{tot}}(\vec{a}) = 2$°C

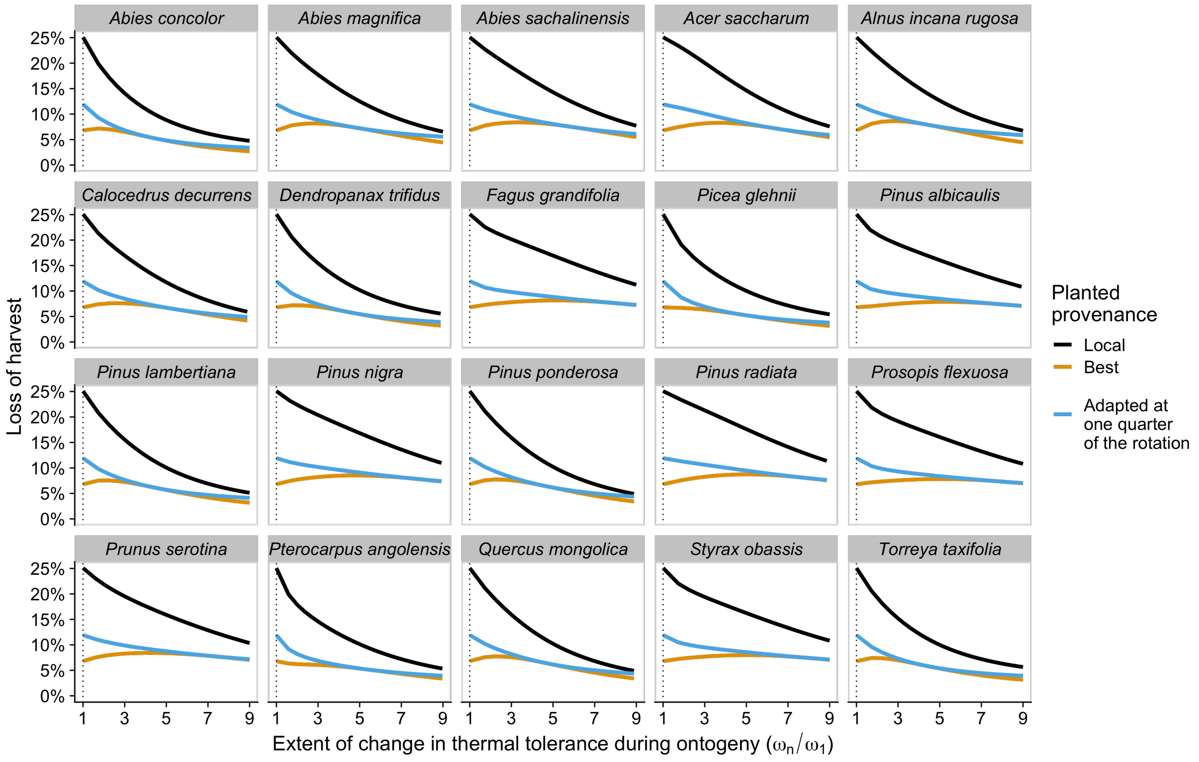

Distributed under a Creative Commons Attribution — 4.0 International licence

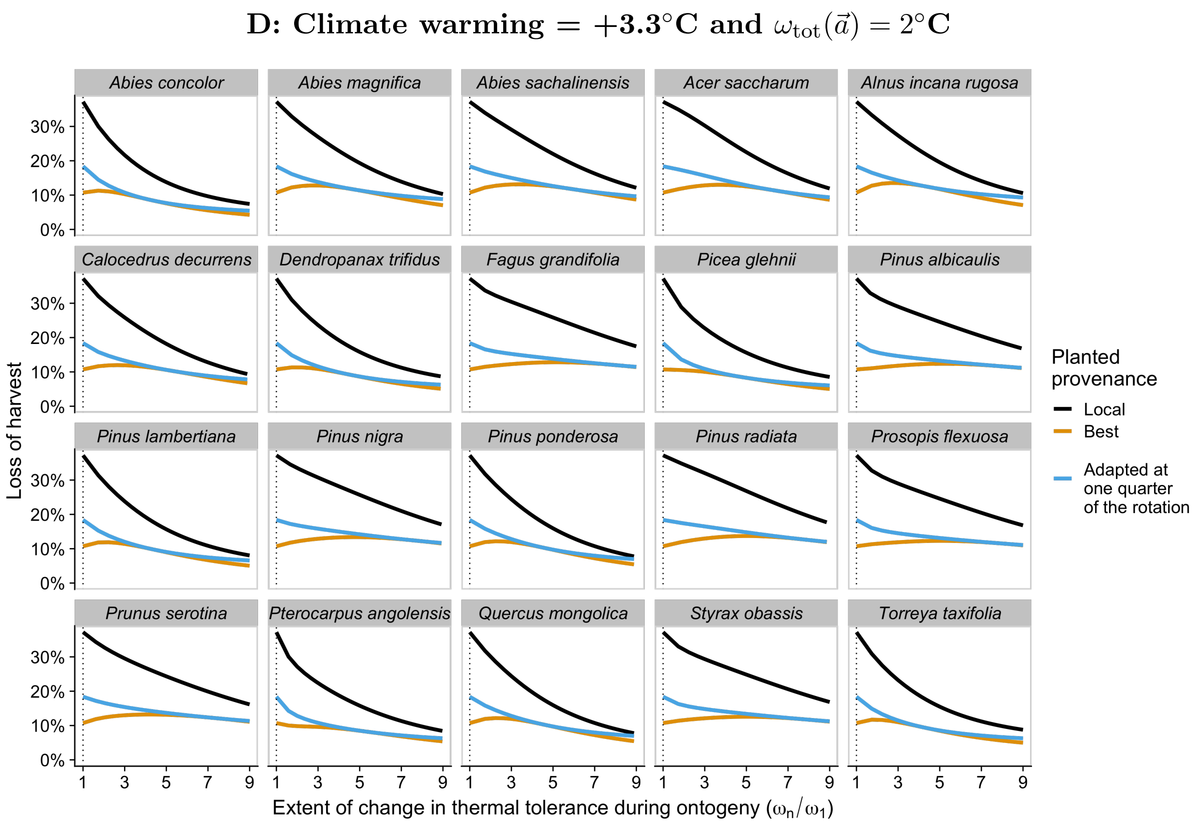

Suppl. Fig. 7: Modifying both the cumulative thermal tolerance ($\omega_{tot}(\vec{a})$) and the strength of climate warming has a major impact on the percentage of loss of harvest - increasing with worse climate change and lower cumulative thermal tolerance. The shape of the response comparing the seed sources (local, best and adapted to temperature at one quarter of the rotation time), however, remains unchanged in all cases and for all species. For all panels, harvest loss was measured against the performance of the local provenance in a scenario with no climate change. The vertical dotted line indicates the case where tolerance is constant across all stages.

Literature cited

Cotto, O., & Chevin, L.-M. (2020). Fluctuations in lifetime selection in an autocorrelated environment. *Theoretical Population Biology*, *134*, 119–128. https://doi.org/10.1016/j.tpb.2020.03.002

Rehfeldt, G. E., Ying, C. C., Spittlehouse, D. L., & Hamilton Jr, D. A. (1999). Genetic responses to climate in *Pinus contorta*: niche breadth, climate change, and reforestation. *Ecological monographs*, *69*(3), 375–407. https://doi.org/10.1890/0012-9615(1999)069[0375:GRTCIP]2.0.CO;2

Distributed under a Creative Commons Attribution — 4.0 International licence
