# Supplementary material for: Planting long‐lived trees in a warming climate: Theory shows the importance of stage‐dependent climatic tolerance
Source: Evol Appl. 2024 Jun 17;17(6):e13711. doi: 10.1111/eva.13711 (PMC11183180; doi:10.1111/eva.13711)
Supplement: Supplementary file 5 — Code S3. [file EVA-17-e13711-s002.docx]

**Planting long-lived trees in a warming climate: theory shows the importance of stage-dependent climatic tolerance**

Adèle Erlichman, Linnea Sandell, Sarah P. Otto, Sally N. Aitken, Ophélie Ronce

**# Codes:**

## 1. Code S1: Mathematica file (CodeS1.nb)

This code can be used to generate the data files (.xls and .csv) used to produce the figures presented in the paper. The mathematica notebook has all parameter values entered manually and so does not require any other files to run.

*n.b.* Mathematica notebooks can be viewed in the Wolfram Mathematica player which can be downloaded from: <https://www.wolfram.com/player/>

## 2. Code S2: R script (CodeS2.R)

This code processes the .csv and .xls data files computed by the Mathematica file and produces the figures presented in the paper. The first part of the R script « Map (Suppl. Fig. 5) and expected warming at the geographic coordinates of the studied tree species » requires downloading data from the internet (IPCC maps).

**# Detailed HOW TO Mathematica file:**

## To generate all the data files necessary to reproduce the figures, just run the whole code all at once (takes about 48 hours on my computer).

## To generate the files separately, each part (*e.g.*, Analytical results) starts by implementing the useful functions, then the parameters, before running the simulations and exporting simulated data in an output file.

[Analytical results]

- ### To compute the general analytical predictions (Fig. 2) and/or the effect of changes in thermal tolerance across stage and life history on the optimal provenance (Fig. 3) run all the parts in Analytical results (*i.e.*, « Model implementation », « Other necessary functions for runs », « Parameters », « Calculation of the thermal tolerance in each stage » and « Simulations and output for R »)
- [Increasing phenotypic variance at the plantation site]
  - ### To compute the effect of increasing phenotypic variance at the plantation site (Suppl. Fig. 1 and 2) run the following parts from Analytical results: « Model implementation », « Other necessary functions for runs » and « Parameters ». You can then run « Functions », « Effect of increasing phenotypic variance » and/or « Effect of increasing phenotypic variance when the climate fluctuates ».

[Simulation results]

- ### To compute the simulations results with a linear increase of the optimum (Fig. 4, 5 and Suppl. Fig. 3, 7), run « Model implementation » and « Other necessary functions and parameters for runs ». You can then run the « Simulations for [all or just one] omega_tot value ».
- ### To compute the figure with slow-er growing species (Supp. Fig. 6), run « Model implementation » and « Other necessary functions and parameters for runs » first.
- ### To compute the simulations with a fluctuating optimum (Fig. 6), run « Model implementation » and « Other necessary functions and parameters for runs » first.

[Comparing analytical predictions to simulations]

- ### Run the following parts from Analytical predictions: « Model implementation », « Other necessary functions for runs », « Parameters » and the following parts from Simulation results: « Model implementation » and « Other necessary functions and parameters for runs ». Then run « Functions » and « Parameters » before running the « Simulations ».

**# Detailed HOW TO R script:**

Always start by running the set up.

## « Map (Suppl. Fig. 5) and expected warming at the geographic coordinates of the studied tree species » can be computed independently of the other parts. You will require the GeoTIFF maps for the IPCC website. Example: the following file « CMIP6 ## Mean temperature (T) Change deg C - Long Term (2081-2100) SSP2-4.5 (rel. to 1995-2014) - Annual (34 models).tiff » downloaded and renamed as « CMIP645.tiff » in the code.

### General analytical predictions (Fig. 2) and Analytical results (Fig. 3) can each be computed independently of the other parts.

### The following parts: « Checking expected omega_tot with the realized omega_tot », « Optimal provenance for 20 temperate tree species in a warming climate (Fig. 4) », « Comparing the performance of different seed sources (Fig. 5 and Suppl. Fig. 7) », « Climate fluctuations (Fig. 6) » all require that « Downloading files » from « Simulations with vital rates for twenty long-lived temperate tree species » have been run beforehand (and it takes a bit of time to download).

### « Increasing phenotypic variance at the plantation site (Suppl. Fig. 1 and 2) », « Comparing analytical results and simulations (Suppl. Fig. 4) » and « Longer rotation time (Suppl. Fig. 6) » can each be computed independently of the other parts.
